# Landscape of plastid DNA breaks in Arabidopsis during development and environmental stimulus

**DOI:** 10.1093/plcell/koag186

**Published:** 2026-06-16

**Authors:** Wenjie Wang, Chengxia Zheng, Kuan Li, Qianwen Sun

**Affiliations:** Vegetable Genetics and Breeding Laboratory, Anhui Province Key Laboratory of Horticultural Crop Quality Biology, School of Horticulture, Anhui Agricultural University, Hefei, China; State Key Laboratory of Green Biomanufacturing, and Center for Plant Biology, School of Life Sciences, Tsinghua University, Beijing, China; Tsinghua–Peking University Center for Life Sciences, Beijing, China; State Key Laboratory of Green Biomanufacturing, and Center for Plant Biology, School of Life Sciences, Tsinghua University, Beijing, China; China Center of Industrial Culture Collection, China National Research Institute of Food and Fermentation Industries Co., LTD, Beijing, China; State Key Laboratory of Green Biomanufacturing, and Center for Plant Biology, School of Life Sciences, Tsinghua University, Beijing, China; Tsinghua–Peking University Center for Life Sciences, Beijing, China

## Abstract

External and internal environmental factors can lead to DNA breaks. DNA breaks in eukaryotic nuclear genomes have been extensively studied, but the landscape of DNA breaks in endosymbiotic organelles remains poorly understood. Here, we employed DNA end tailing and sequencing to profile DNA breaks in the Arabidopsis (*Arabidopsis thaliana*) plastid genome. We find that plastid DNA in cotyledons and true leaves remains relatively stable during the juvenile stage but undergoes extensive breaks in older tissues. Seeds had significantly lower break levels than leaves. Notably, ribosomal DNA regions experience more breaks, with a preference for the template strand. We further investigated plastid DNA break profiles in wild-type (Col-0) plants vs mutants defective in DNA repair, replication, and transcription under varying light, temperature, and photoperiod conditions. Wild-type plants maintained genome integrity under most tested conditions, but the plastid DNA damage repair and replication mutants *why1*/*3*/*reca1* (lacking *Whirly1* (*WHY1*), *WHY3*, and *RECA1*), *why1*/*3*/*polIb* (lacking *WHY*1/*3* and *DNA polymerase IB*), and *atrnh1c* (lacking *RNase H1C*), did not. Additionally, the accumulation of R-loops and reactive oxygen species (ROS) potently induces plastid DNA breaks that are likely mediated by 8-oxoG modifications. Shortening the photoperiod alleviates plastid DNA breaks in Col-0, with an even more pronounced effect in *atrnh1c*. This study provides a genome-wide view of plastid DNA break dynamics and advances our understanding of damage and repair mechanisms in the organellar genome.

## Teaser

The integrity of the plastid genome declines with plant development, and low temperature and short photoperiod could alleviate ptDNA breaks.

## Introduction

DNA serves as the most critical genetic material for cellular processes, and the maintenance of its stability is fundamental to ensuring the proper growth and development of organisms. However, throughout the lifecycle of an organism, its genomic DNA is continuously exposed to a variety of external and internal deleterious factors. These include ionizing radiation, ultraviolet radiation, high temperatures, heavy metals, peroxides, replication arrest, and false replication, and others, all of which can induce various forms of DNA damage (Mingard et al. 2020). Such damage encompasses base loss or insertion, base modification, inter-chain crosslinking, intra-chain crosslinking, DNA–protein crosslinking, single-strand breaks (SSBs), and double-strand breaks (DSBs) (Tubbs and Nussenzweig 2017; Yousefzadeh et al. 2021). Among these, DSBs are the most severe form of damage, as they can lead to genomic instability, chromosomal aberrations, and even cell death if not adequately repaired (Smeenk and van Attikum 2013; Tubbs and Nussenzweig 2017). Importantly, all other types of DNA damage have the potential to ultimately result in DSBs (Waterman et al. 2020).

In addition to the nuclear genome, eukaryotes possess semi-autonomous organellar genomes, including the mitochondrial genome in both animals and plants, and the plastid genome in plants. Mitochondria and plastids are believed to have evolved from endosymbiotic bacteria (Archibald 2015). Their chromosomes are condensed in nucleoids that reside in the membrane and exhibit prokaryotic-like properties (Kucej and Butow 2007; Powikrowska et al. 2014). The organellar genomes primarily encode proteins involved in respiration, photosynthesis, and the regulation of gene expression (Daniell et al. 2016; Zardoya 2020). However, these organelles are also major sources of damaging by-products, as electrons leaking from the respiration and photosynthetic electron transport chains drive the generation of highly reactive oxidants (Waszczak et al. 2018). Consequently, organellar genomes are particularly vulnerable to damage.

The damage to plastid DNA (ptDNA) in plants has been studied for decades, with various methods employed to assess the extent of DNA breaks. The most widely used techniques for evaluating the molecular integrity of ptDNA rely on the electrophoretic mobility of DNA molecules, including DNA Movies, restriction analysis, pulsed-field gel electrophoresis (PFGE), and comet assay (Bendich 1991; Rowan et al. 2010; Greiner et al. 2020; Wang et al. 2021). Additionally, the stability of the plastid genome can be assessed through buoyant density analysis of single-stranded and double-stranded ptDNA using analytical CsCl equilibrium gradients (Greiner et al. 2020). Recently, we applied the terminal-deoxynucleotidyl transferase-mediated nick end labeling (TUNEL) assay to detect the DNA breaks in chloroplasts (Yang et al. 2020; Wang et al. 2021). Furthermore, standard qPCR and long-range PCR assays have been used in combination to assess ptDNA damage in maize. This approach is based on the premise that DNA breaks or modifications can inhibit amplification by *Taq* DNA polymerase during long-range PCR but have minimal impact on qPCR (Kumar et al. 2014). However, inconsistent results have been reported by different research groups (Kumar et al. 2014; Ma and Li, 2015).

A limitation of these methods is that they only provide an overall measurement of ptDNA break levels, without revealing the specific locations or frequencies of breaks. Moreover, despite extensive research on the fragmentation and degradation of organellar DNA (orgDNA) during plant development, the findings remain highly controversial (Oldenburg and Bendich 2015; Greiner et al. 2020). For instance, Bendich and colleagues demonstrated a drastic decline in the molecular integrity of orgDNA during leaf development (Oldenburg and Bendich 2015), whereas Greiner and colleagues argued that plastid genomes in mesophyll tissues remain remarkably stable until senescence (Greiner et al. 2020). Consequently, there is a pressing need for precise methods to measure the loci and frequency of DNA breaks along organellar genomes. Such advancements would not only enhance our understanding of orgDNA stability but also shed light on the mechanism of DNA damage, repair, and replication in plant endosymbiotic organelles.

Over the last decade, a growing number of high-throughput sequencing methods have been developed for genome-wide profiling of DNA breaks. BLESS pioneered the direct mapping of DNA breaks (Crosetto et al. 2013). Subsequent refinements gave rise to methods with improved efficiency and quantitative capability, such as END-seq (Canela et al. 2016), DSB-capture (Lensing et al. 2016), BLISS (Yan et al. 2017), i-BLESS (Biernacka et al. 2018), qDSB-seq (Zhu et al. 2019), TrAEL-seq (Kara et al. 2021), and INDUCE-seq (Dobbs et al. 2022). In parallel, methods such as SSiNGLe (Cao et al. 2019) and GLOE-Seq (Sriramachandran et al. 2020) have enabled the mapping of SSBs. However, the application of these methods has been largely confined to nuclear genomes, especially mammalian and yeast, leaving the landscape of DNA breaks in semiautonomous organellar genomes largely unexplored.

Recently, we developed a technique, named DNA end tailing and sequencing (DEtail-seq) (Xu et al. 2023). Unlike the methods mentioned above, DEtail-seq requires neither thermal denaturation (which risks introducing additional breaks), nor end blunting (which may cause further damage or artifactual breaks), nor biotin–streptavidin enrichment (whose efficiency can vary substantially across samples). Omission of these steps substantially streamlines the protocol (Table S1). DEtail-seq employs a commercial enzyme complex (Adaptase) to directly ligate an adapter to extended 3′ DNA ends, enabling the entire library preparation to be completed within 2 d. Consequently, it is primarily optimized for the direct and highly efficient capture of DSBs bearing 3′ overhangs (Xu et al. 2023; Zhang et al. 2024). This method has been successfully applied across a range of species, including yeast, mouse, human, and plant (Xu et al. 2023; Zhang et al. 2024). In this study, we adapted DEtail-seq to investigate the distribution of DNA breaks in the plastid genome of Arabidopsis. We conducted a comprehensive analysis to decipher the landscape of ptDNA break in leaves at different seedling ages, as well as in various other tissues. Additionally, we examine the dynamics of ptDNA breaks in mutants with defects in DNA repair, replication, and transcription under various environmental conditions. Our results reveal that ptDNA breaks increase as plants develop, and loss of DNA replication and repair factors leads to DNA instability.

## Results

### Genome-wide ptDNA break mapping by DEtail-seq

**Figure 1:**
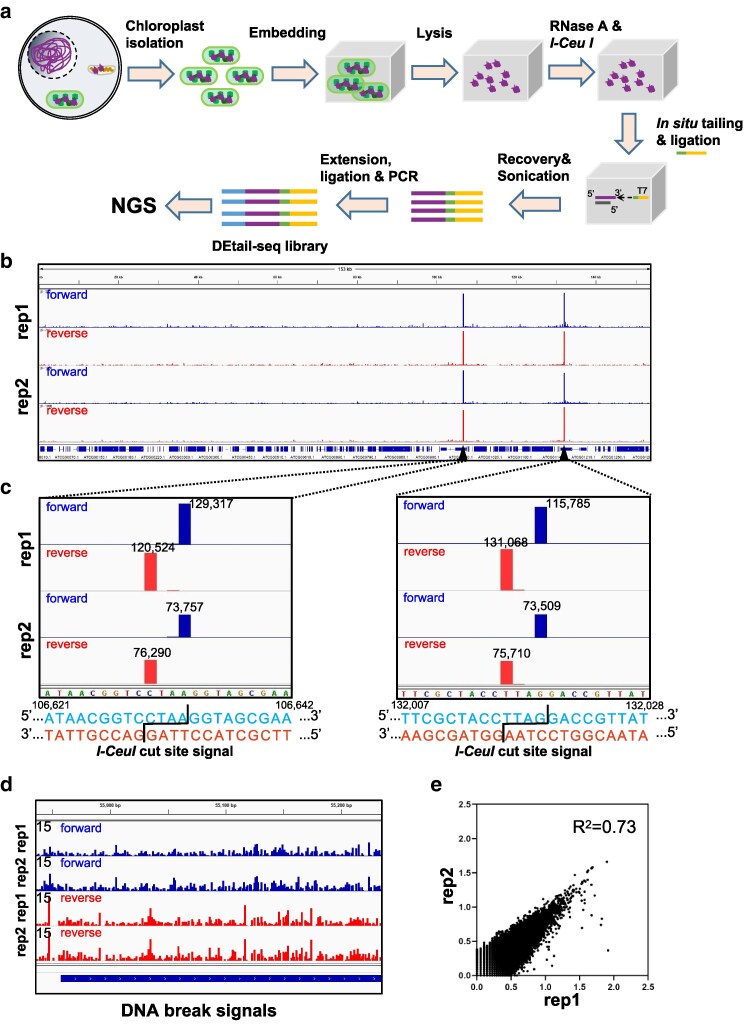
DEtail-seq precisely and efficiently maps ptDNA breaks. a) Schematic overview of the DEtail-seq method. Chloroplasts were isolated from tissues, embedded in agarose plugs, and lysed. An ssDNA adapter was then ligated to the 3′ overhangs of DSBs in situ. After denaturation, recovery, and sonication of DNA, the DEtail-seq library was constructed by completing the remaining steps of the dedicated ssDNA library preparation kit. b) Genome browser profiles of artificial DSBs generated by *I-CeuI* cleavage. Raw sequencing read counts are displayed directly. Black triangles indicate the *I-CeuI* cutting sites. c) Enlarged view of (b). Top: Mapping of DEtail-seq reads at the cutting sites on the forward and reverse strands. The number of reads is displayed above the bars. Bottom: Sequence and loci of *I-CeuI* sites. Broken lines indicate the break ends. d) A representative snapshot of 2 biological replicates demonstrates the reproducibility of DEtail-seq. Methods for data processing are described in the “Sequencing data processing” section. e) Correlation of DEtail-seq signals between 2 biological replicates on the forward strand. Normalized read counts (log_10_(*n* + 1)) were compared at every single base, and Pearson's correlation coefficient (*R*^2^) is indicated.

A detailed workflow of the modified DEtail-seq method for ptDNA is depicted in Fig. 1a. Briefly, plastids were isolated from fresh tissues and immediately embedded in low-melting-point agarose. Subsequent steps, including plastid lysis, RNA digestion, *I-CeuI* treatment, and single-strand DNA (ssDNA) adaptor ligation, were performed in situ to minimize artificial DNA breaks. Following 3′ overhang tailing and ligation, the DNA was recovered from the agarose, subjected to extension, 5′ end truncated adaptor ligation, and PCR amplification.

To enable quantitative measurement of DNA break levels, spike-in DNA breaks were introduced by digesting the plastid genome in agarose before tailing and ligation steps, using the rare-cutting restriction enzyme *I-CeuI* (Fig. 1a). The *I-CeuI* digestion generates 3′ extended ends and has only 2 recognition sites in the plastid genome (Fig. 1b and c). To assess the digestion efficiency, we performed qPCR to compare the integrity of 3 distinct genomic regions: the *I-CeuI* cutting site itself, an adjacent region, and a distal region (Figure S1a). Following *I-CeuI* treatment, the integrity at the cut site dropped to <0.5% of the level observed in uncut controls (Figure S1a), demonstrating near-complete digestion with an efficiency exceeding 99.5%.

To validate the reliability of the DEtail-seq method, we prepared libraries using chloroplasts isolated from 2-week-old Col-0 seedlings with 2 biological replicates. Sequencing data were processed through a custom bioinformatic pipeline to remove the A-tail, map the reads, and deduplicate them. Clean reads were then mapped to the Arabidopsis Information Resource (TAIR10) genome. Over 93% of reads from each sample mapped to the plastid genome (Figure S1b), and DEtail-seq signals were highly enriched at the 3′ termini of spike-in DNA breaks (Fig. 1b and c), accounting for more than 15% of the total reads (Figure S1b). By excluding reads at *I-CeuI* sites and normalizing the data to spike-in sites (see method for details), we observed highly consistent break patterns between the 2 biological replicates in both strands (Fig. 1d and Figure S1c). Furthermore, the Pearson correlation coefficients between the replicates were strong (Fig. 1e and Figure S1d), indicating that the results were highly reproducible and the detected signals correspond to genuine DNA break events rather than background noise. Together, these data demonstrated that our single-nucleotide break mapping method is efficient, precise, and reproducible in profiling ptDNA breaks.

### ptDNA breaks in the leaf increase along with seedling age

**Figure 2:**
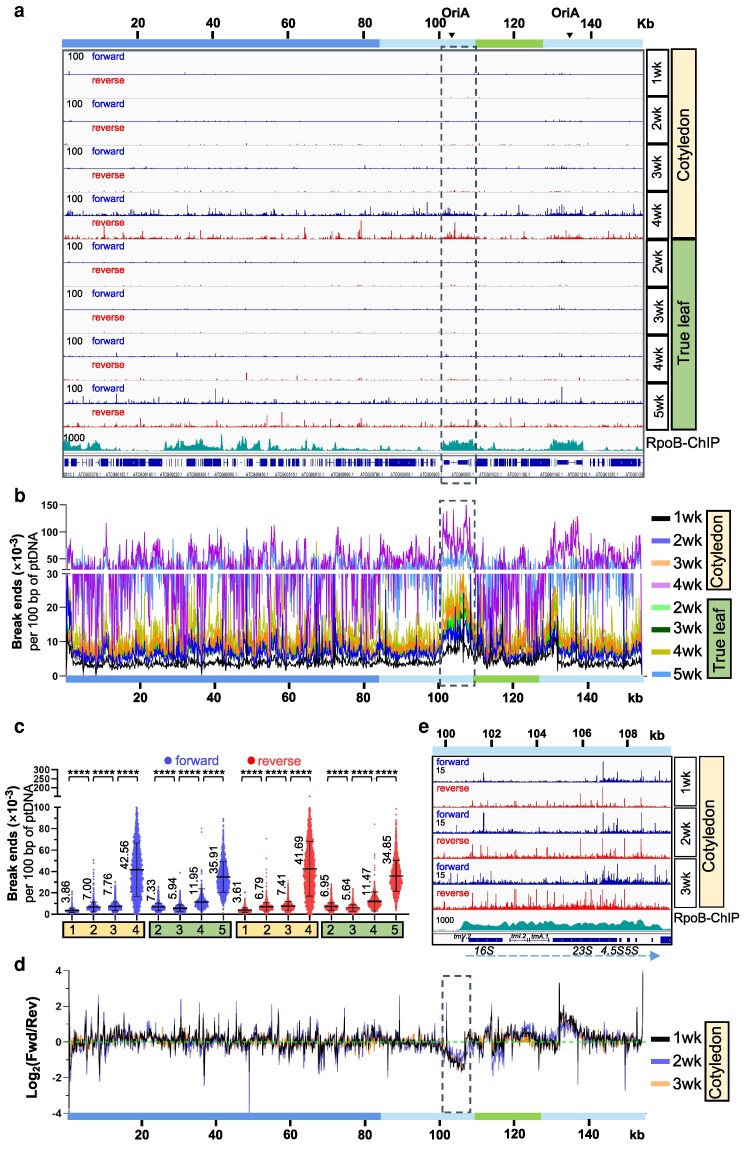
Profiling of ptDNA breaks in cotyledons and true leaves at different developmental stages. a) Overview of the ptDNA break patterns in cotyledons from 1-, 2-, 3-, and 4-week-old seedlings and in freshly emerging true leaves from 2-, 3-, 4-, and 5-week-old seedlings. Data range in each IGV track is 0 to 100. b) Comparison of DNA break intensity on the reverse strand across the plastid genome in the indicated samples. The signal was summed in consecutive, nonoverlapping 100-bp windows across the genome to generate the line plot. c) Number of sequenced break ends mapped per 100 bp along the plastid genome. Central line: mean (values shown adjacent to scatter points). Error bars: standard deviation (SD). Asterisks: Wilcoxon matched-pairs signed rank test, ****P < 0.0001. *x* axis: weeks. d) Comparison of break intensity between 2 DNA strands in cotyledons from 1-, 2-, and 3-week-old seedlings. The ratio of break ends per 100 bp on the forward strand to that on the reverse strand is shown on a log_2_ scale. Fwd: forward, Rev: reverse. e) Enlarged view of the rDNA region outlined by the black-dotted box in (a), containing the *trnV.2*, *16S*, *trnI.2*, *trnA.1*, *23S*, *4.5S*, and *5S* genes. Data ranges are indicated. Arrowheads denote transcriptional direction. RpoB ChIP-seq data is included for reference. A schematic map illustrates the organization of the plastid genome: large single-copy region (LSC, dark blue bar), a small single-copy region (SSC, green bar), and 2 inverted repeats (IRs, light blue bars). OriA indicates replication origin. The dotted box outlines the rDNA region.

We then applied DEtail-seq to investigate how ptDNA break patterns change across different seedling ages. Leaf tissues were harvested from Arabidopsis plants at 1, 2, 3, 4, and 5 wks of age (Figure S2a). First, we compared ptDNA breaks in cotyledons from 1-, 2-, 3-, and 4-week-old plants, as well as in newly emerged true leaves from 2-, 3-, 4-, and 5-week-old plants (Figure S2b). While ptDNA in cotyledons remained relatively stable at Weeks 1 to 3, strong DEtail-seq signals were detected at Week 4 (Fig. 2a and Figure S3a), coinciding with the onset of rapid senescence of cotyledons. In newly emerged true leaves, DEtail-seq signals were low in 2- and 3-wk-old seedlings, similar to levels in cotyledons, but increased significantly at Week 4 and further rose at Week 5 (Fig. 2a and Figure S3a).

To quantitatively compare DNA break intensity along the plastid genome across samples, the normalized DEtail-seq signal at each base was summed in consecutive, nonoverlapping 100-bp windows. When displayed on the forward strand, break intensity increased across nearly the entire plastid genome in both cotyledons and true leaves as development proceeded (Fig. 2b). On average, a 1-week-old cotyledon showed 3.86 × 10^−3^ break ends per 100 bp, indicating approximately 6 break ends per reverse strand of the plastid genome. This level increased about 2-fold in 2- and 3-week-old cotyledons and surged about 10-fold by Week 4 (Fig. 2c). In newly emerged true leaves, break intensity was similar at Weeks 2 and 3 but increased approximately 2-fold at Week 4 and 5-fold at Week 5 (Fig. 2c). A similar increasing trend was observed on the reverse strand (Fig. 2c). Notably, the average break intensity was comparable between the 2 strands in each sample. Since a DSB should generate one signal on each strand, the similar break intensity between strands suggests that the DEtail-seq signals primarily originate from DSBs. To test this hypothesis, we compared break intensity between the 2 strands across the genome in 100-bp windows, and the results indicated the ratio of forward-to-reverse strand break intensity is close to 1 across almost the entire genome at all stages (Fig. 2d, Figures S3b and c), supporting the prevalence of DSBs.

We observed a notable enrichment of DEtail-seq signals in rDNA regions, particularly in younger tissues (Fig. 2b and Figure S3a). This finding aligns with our previous report that plastid rDNA regions are prone to fragility due to frequent transcription–replication conflicts (TRCs) (Yang et al. 2020; Zhang et al. 2024). To further investigate this, we analyzed recently published ChIP-seq data of RpoB (Palomar et al. 2022), a key component of plastid-encoded RNA polymerase (PEP) involved in transcription at rDNA regions. The results indicated that the intensive DEtail-seq signals were precisely localized within rDNA regions where RpoB was significantly enriched (Fig. 2a and Figure S3a). Furthermore, surprisingly, breaks in rDNA regions were asymmetrically distributed between the 2 DNA strands (Fig. 2d and Figure S3c), with a higher level on the template strand than on the coding strand (Fig. 2e and Figure S3d). On average, the break intensity on the template strand was approximately 1.5 times greater than that on the coding strand at most stages (Figure S3e), while the break intensity on the coding strand in rDNA regions was similar to the genome-wide average (Fig. 2c and Figure S3e). This asymmetry was less pronounced in 4-week-old cotyledons, likely because the high density of breaks in senescent tissues obscured the pattern (Figure S3b). Collectively, our data indicated that DSB frequency increases during seedling development and that rDNA regions are break-prone, with breaks occurring more frequently on the template strand.

### Profiling of ptDNA break patterns in different tissues

**Figure 3:**
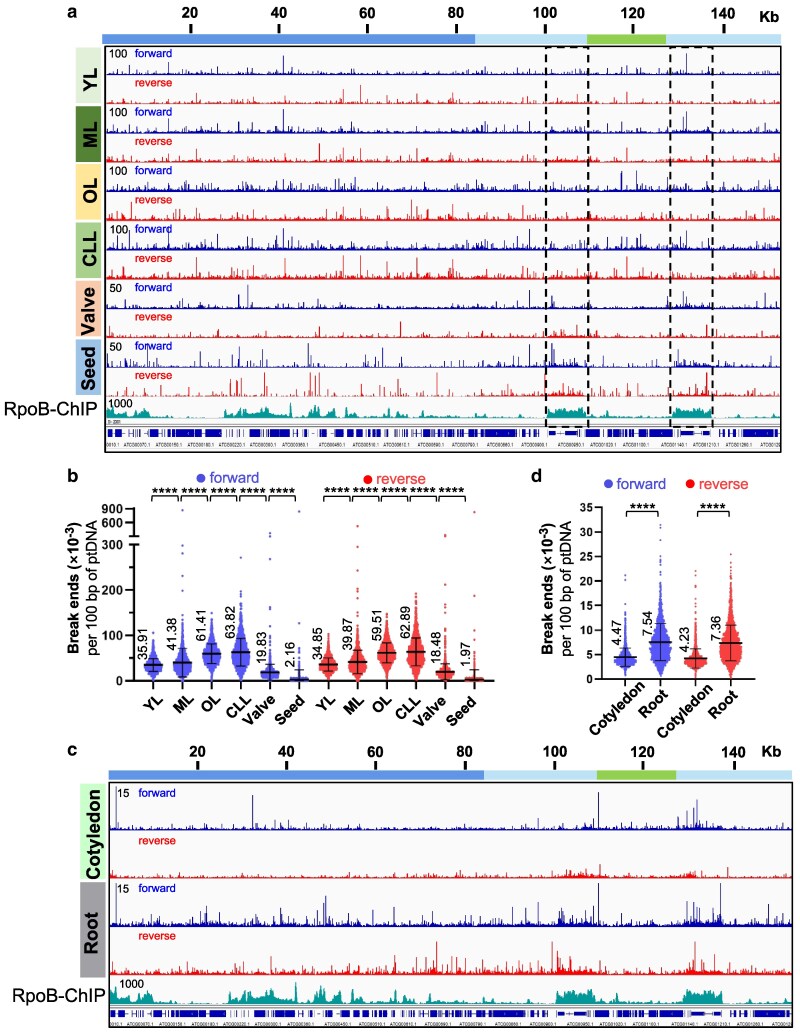
Profiling of ptDNA breaks in different tissues. a) Overview of ptDNA break patterns in various tissues from 5-week-old seedlings. YL, young leaf; ML, mature leaf; OL, old leaf; CLL, cauline leaf. The data range in each IGV panel is 0 to 500. RpoB ChIP-seq data is included. b) Number of sequenced break ends mapped per 100 bp along the plastid genome across the indicated tissues. c) Overview of ptDNA break patterns in root and cotyledon from 1-week-old seedlings. The data range in each IGV panel is 0 to 180. d) Comparison of the ptDNA break level in samples indicated in (c). Central line: mean (values shown adjacent to scatter points). Error bars: SD. Asterisks: Wilcoxon matched-pairs signed rank test, ****P < 0.0001.

Plastids perform diverse functions across tissues, which are partially dependent on the proper expression of the plastid genome (Jarvis and Lopez-Juez 2013; de Souza et al. 2017). Consequently, the integrity of the plastid genome may vary among different tissues. However, the patterns of ptDNA breakage in various organs have rarely been investigated. To address this, we aimed to compare ptDNA break patterns across different tissues. Plastids were isolated from young, mature, and old leaves, as well as cauline leaves, valves, and seeds of 5-week-old plants (Figure S2c), and subjected to DEtail-seq profiling. Overall, the level of ptDNA breaks in young leaves was comparable to that in mature leaves, but significantly increased in old leaves (Fig. 3a and b). Notably, ptDNA broke even more pronouncedly in the first cauline leaf (Fig. 3a and b). In contrast, ptDNA in valves was relatively stable compared to leaves, and it was even more intact in seeds. Among all tissues examined, DEtail-seq signals were most prominently enriched around rDNA regions in seeds, which also coincided with the significant enrichment of RpoB (Fig. 3a). However, the asymmetric break pattern observed in these samples was less pronounced than in the fresh emerging true leaves of 2- and 3-week-old seedlings (Figure S4a), with breaks on the template strand being less than 1.5 times as frequent as the coding strands in rDNA regions (Figure S4b). Intriguingly, in seeds, frequencies were nearly equal between the template and coding strands (Figure S4b). Correlation analysis further indicated that the ptDNA break profile in seeds is poorly correlated with those of other tissues, confirming a distinct break pattern (Figure S4c).

Furthermore, we also assessed ptDNA breaks in the roots of 1-week-old seedlings. The normalized ptDNA breaks in the root averaged approximately 10 breaks per genome, slightly higher than that in cotyledons (Fig. 3c and d). Moreover, the break pattern in the root closely resembles that observed in the leaves (Figure S4c).

### Comparing ptDNA break patterns in DNA repair mutants

In previous studies, we identified AtRNH1C (*Arabidopsis thaliana* RNase H1 C) as a critical factor in maintaining plastid genome stability (Yang et al. 2017). Furthermore, AtRNH1C collaborates with RecA1, Whirly1 (WHY1), and WHY3 in ptDNA repair (Wang et al. 2021). The integrity of ptDNA in mutants lacking AtRNH1C, either alone or in combination with the DNA recombinase RecA1 or the ssDNA binding proteins WHY1/3, had been extensively assessed using several methods, including PFGE, comet assays, and TUNEL assays. These analyses consistently revealed severe genome fragmentation in these mutants (Yang et al. 2017; Wang et al. 2021). However, obtaining a genome-wide map of DNA breaks in these mutants would greatly enhance our understanding of DNA damage and repair processes in plant organelles. To elucidate the role of these genes in ptDNA stability and to further demonstrate the utility of our DEtail-seq method, we constructed and analyzed the DEtail-seq libraries in these mutants.

**Figure 4:**
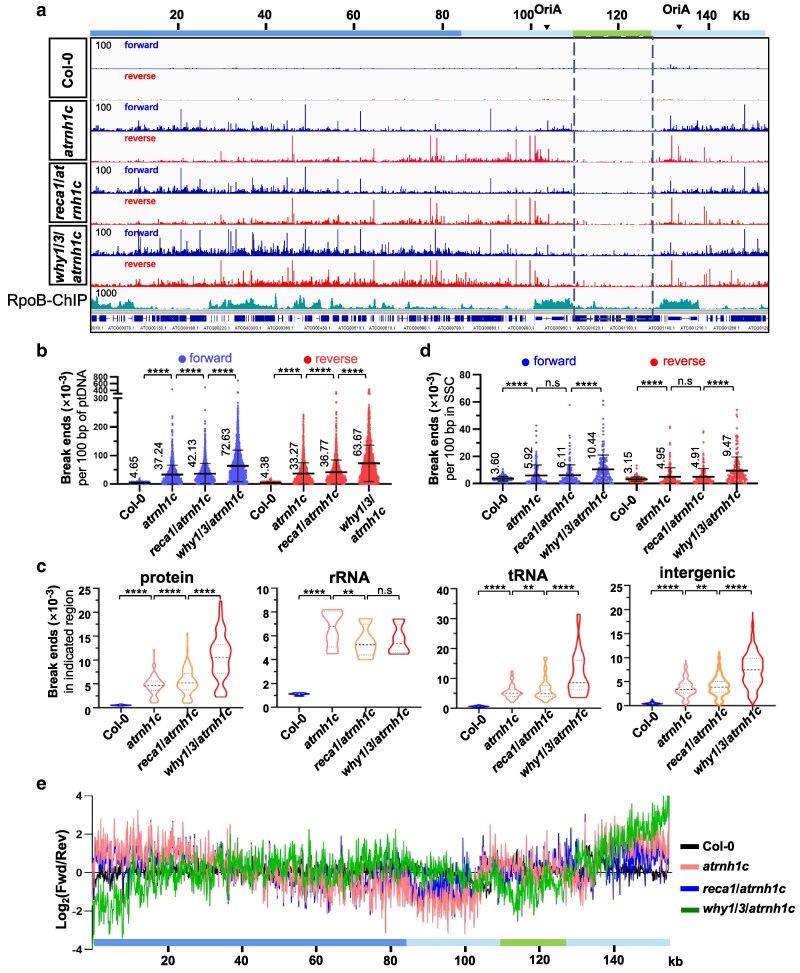
Comparison of ptDNA break patterns in mutants with impaired plastid genome integrity. a) Overview of the ptDNA break patterns in 3-week-old seedlings of Col-0, *atrnh1c*, *reca1*/*atrnh1c*, and *why1*/*3*/*atrnh1c*. The dotted box highlights the SSC region. b) Number of sequenced break ends per 100 bp along the plastid genome. c) Number of sequenced break ends in each gene or intergenic region. The central line indicates the median, and the lower and upper lines represent the first and third quartiles, respectively. d) Number of sequenced break ends mapped per 100 bp in the SSC region. Numbers adjacent to scatter points represent mean values. e) Comparison of break intensity between 2 DNA strands in seedlings of Col-0, *atrnh1c*, *reca1*/*atrnh1c*, and *why1*/*3*/*atrnh1c*. The ratio of break ends per 100 bp on the forward strand to that on the reverse strand is shown on a log_2_ scale. Fwd: forward, Rev: reverse. Asterisks indicate statistically significant differences (Mann–Whitney *U* test for (b) and (d); Wilcoxon matched-pairs signed rank test for (c), **P < 0.01, ****P < 0.0001). n.s., not significant. In (b) and (d), the central line indicates the mean, and error bars represent SD.

We analyzed ptDNA fragmentation in 3-week-old seedlings of Col-0, *atrnh1c*, *reca1*/*atrnh1c*, and *why1*/*3*/*atrnh1c*. While *reca1*/*atrnh1c* exhibited ptDNA break levels comparable to *atrnh1c*, the *why1*/*3*/*atrnh1c* displayed much stronger ptDNA fragmentation in the PFGE assay (Figure S5a and b). Consistent with these findings, the DEtail-seq signal was notably enhanced in *atrnh1c* compared to Col-0 (Fig. 4a). Furthermore, the ptDNA break intensity increased about 8-fold on both forward and reverse strands in *atrnh1c* compared to Col-0 (Fig. 4b). Similarly, the break level in *reca1*/*atrnh1c* was close to that in *atrnh1c*, while *why1*/*3*/*atrnh1c* exhibited the strongest signals, with approximately 16-fold more breaks than Col-0 (Fig. 4a and b). Further analysis revealed that DEtail-seq signals are significantly increased in both genes and intergenic regions (Fig. 4c). Interestingly, the ptDNA breaks were increased in almost all the genomes except in the SSC region (Figure S6a), where *atrnh1c* and *reca1*/*atrnh1c* showed less than 2-fold increase in both the forward and reverse strands compared to Col-0 (Fig. 4d). In *why1*/*3*/*atrnh1c*, the frequency of breaks in SSC was approximately 3-fold that of Col-0, which is significantly lower than the increase observed in other regions (Fig. 4b and d).

Notably, the asymmetric break region was markedly extended in *atrnh1c*, *reca1*/*atrnh1c*, and *why1*/*3*/*atrnh1c*. The forward strand exhibited stronger breaks in the first 40 kb, and the reverse strand broke more at the region of 60 to 100 kb (Fig. 4e and Figure S6b) in *atrnh1c*, *reca1*/*atrnh1c*, while *why1*/*3*/*atrnh1c* showed stronger breaks at the first 40 kb, suggesting an accumulation of single-strand ends in these mutants. However, there was no clear preference for breaks on either the coding or template strands (Figure S6c). Furthermore, Spearman's rank correlation coefficient analysis revealed that the ptDNA break patterns are significantly altered in the mutants compared to Col-0 (Figure S6d).

Given that AtRNH1C plays a role in maintaining plastid genome integrity by finely regulating the level of R-loop levels—a secondary DNA structure composed of a DNA:RNA hybrid strand and a single DNA strand (Yang et al. 2017), we asked whether the break-silent valley (BSR) is due to the insufficient R-loop accumulation in the SSC region. To explore this, we analyzed R-loop deposition in the plastid genome using ssDRIP-seq, a high-throughput sequencing method for genome-wide R-loop profiling (Xu et al. 2017). The results showed that R-loops level is elevated in *atrnh1c* across nearly the entire LSR and IRs, but only marginally increases in the SSC region (Figure S7a). These patterns are parallel with the binding profile of PEP RpoB (Palomar et al. 2022).

**Figure 5:**
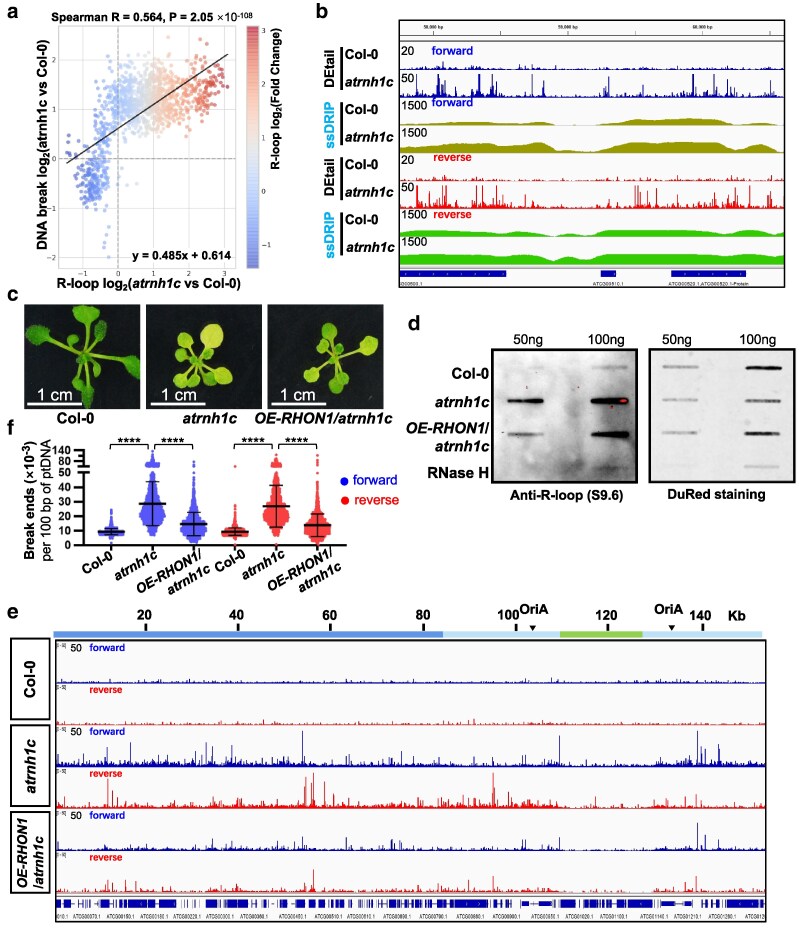
Accumulation of R-loops leads to increased ptDNA break. a) Genome-wide correlation between R-loop accumulation and plastid DNA break frequency. Scatter plot showing the positive relationship between R-loop log_2_ (*atrnh1c* vs Col-0) fold change, and DNA break log_2_(*atrnh1c* vs Col-0) fold change across the plastid genome (Spearman's *R* = 0.564, *P* = 2.05 × 10^−108^). Each point represents a 100-bp genomic window. b) Genome-browser snapshots of a representative region showing co-increased R-loops (ssDRIP-seq) and DNA breaks (DEtail-seq). c) Phenotypes of 3-week-old seedlings of Col-0, *atrnh1c*, *OE-RHON1*/*atrnh1c*. d) Slot-blot analysis of total R-loops levels in ptDNA from the genotypes indicated. DNA treated with RNase H was included as a control. R-loops were detected using the S9.6 antibody (left panel); DuRed staining shows equal DNA loading (right panel). e) Genome-wide profiles of ptDNA breaks in 3-week-old seedlings of Col-0, *atrnh1c*, *OE-RHON1*/*atrnh1c*. f) Quantitative comparison of DNA breaks, presented as the number of sequenced break ends per 100 bp along the plastid genome. The central line indicates the mean, and error bars represent SD. Asterisks indicate statistically significant differences (Mann–Whitney *U* test, ****P < 0.0001).

To investigate the relationship between R-loop dynamics and DNA break formation in ptDNA, we further compared ssDRIP-seq (R-loop) and DEtail-seq (DNA break) profiles in Col-0 and *atrnh1c* plants. Plastid genome-wide correlation analysis revealed a significant positive association between R-loop accumulation and DNA break frequency (Spearman's *R* = 0.564,  *P* = 2.05 ×10^−108^, Fig. 5a), with numerous local regions visually exemplifying this co-increase pattern (Fig. 5b). To establish a functional—rather than merely correlative—connection, we took a genetic approach. We overexpressed *RHON1*, a known plastid-localized helicase capable of resolving R-loops, in the *atrnh1c* mutant background (Yang et al. 2020). Compared to *atrnh1c*, the *OE-RHON1*/*atrnh1c* line exhibited a nearly normal seedling phenotype (Fig. 5c), a significant reduction in global plastid R-loop levels (Fig. 5d), and, most importantly, a pronounced decrease in overall ptDNA breaks (Fig. 5e and Figure S7b). This genetic intervention, which directly reduces R-loop burden, resulted in a concomitant suppression of DNA break accumulation, supporting a causal role for R-loops in promoting DNA breakage.

### Profiling of ptDNA break patterns in replication and transcription mutants

**Figure 6:**
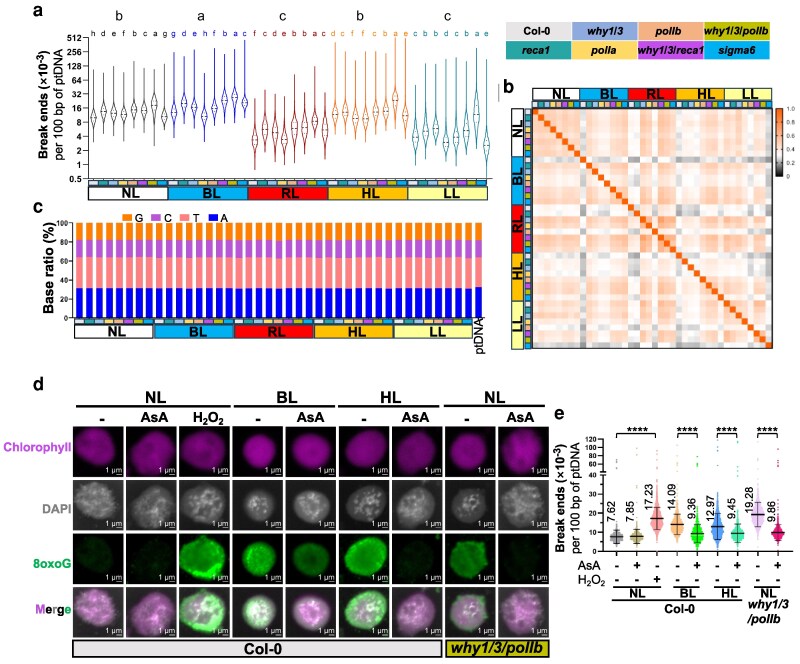
ptDNA break patterns in replication and transcription mutants. a) ptDNA break level in mutants under different light treatments. Number of sequenced break ends mapped per 100 bp along the plastid genome. In the violin plot, bulges indicate higher data density, whiskers denote outliers, central line: median, lower and upper lines: first and third quartiles. Different lowercase letters indicate significant differences, as determined by Dunn's post hoc test (*P* < 0.05). b) Heatmap of Spearman's rank correlation coefficients of DEtail-seq signals from the reverse strand across sample pairs. c) Ratio of break sites at A/T/C/G nucleotides in the plastid genome. d) Immunofluorescence detection of 8oxoG accumulation in chloroplasts. Sixteen-day-old seedlings grown on 1/2 MS medium under normal light were transferred to fresh 1/2 MS medium supplemented with 0.5 mM AsA or 2 mM H_2_O_2_ and cultured for an additional 5 d under the indicated light conditions. e) Comparison of the ptDNA break level in seedlings described in (d). Numbers adjacent to scatter points represent mean values. Asterisks indicate statistically significant differences (Mann–Whitney *U* test, ****P < 0.0001). NL, normal light; BL, blue light; RL, red light; HL, high light; LL, low light.

It has been widely recognized that DNA replication and transcription often occur simultaneously within the same genome, potentially leading to TRCs and subsequent genome instability. In addition to their roles in DNA repair, WHY1/3, RecA1, and the DNA polymerase PolIB are involved in DNA replication and recombination (Parent et al. 2011; Zampini et al. 2015). Notably, PolIA and PolIB are the only 2 known DNA polymerases present in chloroplasts, each with distinct functions (Parent et al. 2011; Zhang et al. 2024). Additionally, chloroplast sigma factors are core subunits of the PEP, which directs transcription (Puthiyaveetil et al. 2021). To investigate the impact of loss of these factors, either individually or in combination, we analyzed the ptDNA damage level under normal light (NL) conditions. The results showed elevated ptDNA damage in *reca1*, *why1*/*3*, *polIa*, and *polIb* single mutants, with further increases observed in *why1*/*3*/*reca1* and *why1*/*3*/*polIb* triple mutants (Fig. 6a). This aligns with the growth phenotype, as *why1*/*3*/*polIb* exhibited the most severe growth defects (Figure S8a). Loss of *SIGMA6* (SIGMA FACTOR 6) had no significant effect on ptDNA stability (Fig. 6a).

The chloroplast is one of the most sensitive cellular compartments in response to environmental changes. Therefore, it is hypothesized that ptDNA stability may vary in response to environmental stimuli. Since light quality and intensity are among the most frequently encountered environmental variables for plants, we examined the ptDNA break pattern in these mutants grown under different light qualities and intensities (Figure S8a). The results showed that ptDNA break levels were reduced under red light (RL) and low light (LL) but increased under blue light for most mutants (Fig. 6a). Under high-light (HL) conditions, break levels remained largely unaffected in most mutants, except for *why1*/*3*/*polIb*, which exhibited increased breaks (Fig. 6a). A previous report had shown that *why1*/*3*/*polIb* had lower Fv/Fm under LL, resulting in reduced production of reactive oxygen species production and consequently a more normal phenotypic performance (Lepage et al. 2013). Thus, the observed reduction in ptDNA breaks in the mutants may correlate with the reduced photosynthetic activity (Figure S8b). Although break levels varied among different mutants in varied conditions, the break patterns were relatively conserved in most mutants under most conditions (Figure S8c). This conservation is further supported by Spearman's rank correlation coefficients, which revealed a moderate similarity (ratio > 0.3) in ptDNA break patterns between most samples (Fig. 6b). Additionally, the ptDNA break sites exhibited a similar nucleotide distribution, with adenine (A) and thymine (T) each accounting for approximately 32%, and cytosine (C) and guanine (G) accounting for approximately 18%. This distribution aligns closely with the overall A/T/C/G bases observed in the plastid genome (Fig. 6c).

8-Hydroxydeoxyguanosine (8-oxoG) is a widespread DNA lesion induced by reactive oxygen species (ROS) and contributes to genome instability (Kumar et al. 2020; Hahm et al. 2022). As chloroplasts function as environmental sensors and are prone to ROS accumulation under unfavorable conditions (Li and Kim 2022), we investigated whether the accumulation of ptDNA breaks is associated with ROS levels and 8-oxoG formation. Consistent with this idea, both blue light and high light treatments promoted ROS accumulation in Col-0 plants (Figure S9a and b). Correspondingly, 8-oxoG levels increased in Col-0 under BL and HL treatments (Fig. 6d). To further validate this correlation, we supplemented Col-0 plants grown under BL and HL with ascorbic acid (AsA) to scavenge ROS. This treatment effectively reduced ROS accumulation (Figure S9a and b), decreased 8-oxoG levels (Fig. 6d), and concurrently alleviated ptDNA breaks (Fig. 6e). Conversely, treating Col-0 seedlings with H_2_O_2_ increased both 8-oxoG accumulation and ptDNA breaks. The *why1*/*3*/*polIb* mutant, which exhibits constitutive photosynthetic defects and ROS accumulation even under normal light conditions (Lepage et al. 2013), also showed elevated 8-oxoG levels (Fig. 6d). Importantly, AsA treatment reduced chloroplast ROS, 8-oxoG accumulation, and ptDNA breaks in this mutant (Fig. 6d and e). In line with these findings, we observed that the ROS levels increased in leaves during plant development (Figure S9c). Together, these results suggest that ROS may be a major cause of ptDNA breaks through the induction of 8-oxoG.

### ptDNA break patterns under temperature and photoperiod stimuli

**Figure 7:**
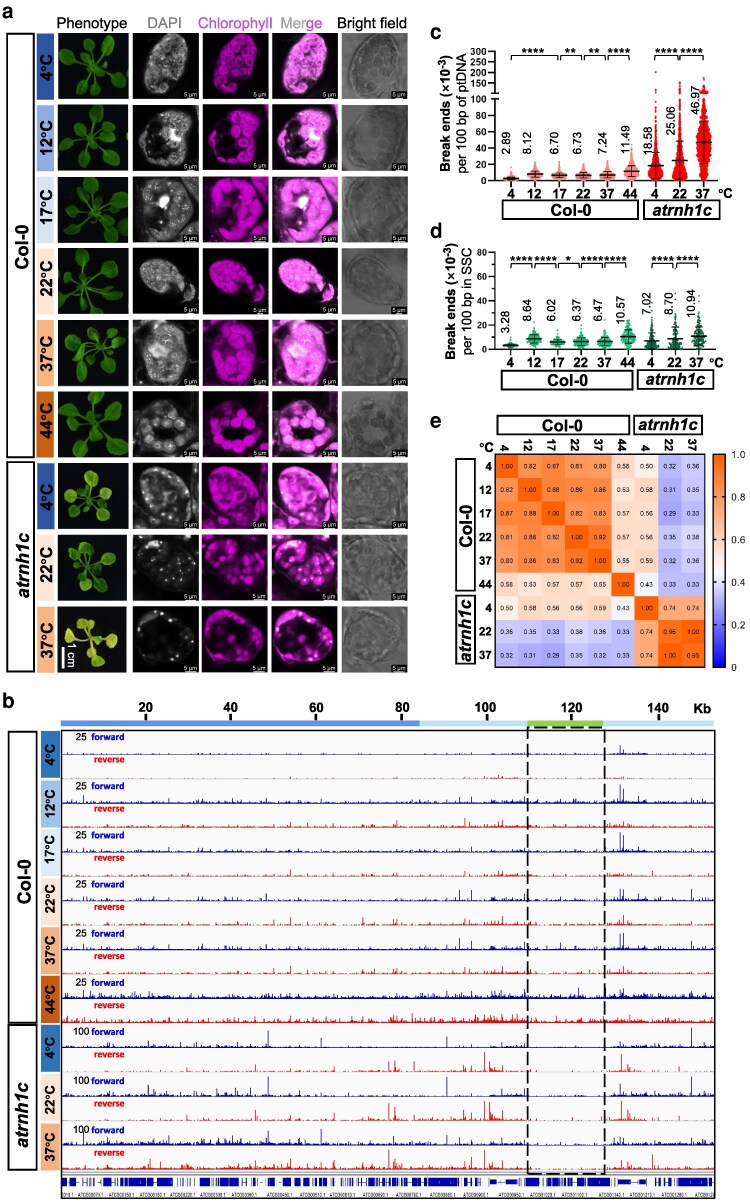
Variation in ptDNA break in response to temperature. a) Morphology of chloroplasts and nucleoids, and seedling phenotypes under different temperature treatments. Seedlings were grown at 22 °C under long-day conditions for 19 d, then transferred to a growth chamber set at different temperatures (4, 12, 17, 22, and 37 °C) for an additional 2 d. For the 44 °C treatment, 21-day-old seedlings were exposed to this temperature for 5 h. All samples were harvested on the same day. b) Overview of ptDNA break patterns in seedlings is indicated in (a). c) Number of sequenced break ends mapped per 100 bp along the plastid genome. d) Number of sequenced break ends mapped per 100 bp in the SSC region. e) Heatmap of Spearman's rank correlation coefficients between pairs of samples. Numbers adjacent to scatter points represent mean values. Central line: mean (values shown adjacent to scatter points). Error bars: SD. Asterisks: Wilcoxon matched-pairs signed rank test, *P < 0.05, **P < 0.01, ****P < 0.0001.

Temperature variation is another critical environmental factor frequently encountered by chloroplasts. Whether chloroplasts can maintain genome stability under such conditions is therefore an important question. To address this, 19-day-old seedlings grown at 22 °C were transferred to growth chambers set at different temperatures (Fig. 7a) and cultivated for an additional 2 d. For the 44 °C treatment, seedlings were grown for only 5 h before leaf wilting occurred. Subsequently, ptDNA breaks were profiled in the samples. The temperature treatments had no significant impact on chloroplast integrity or nucleoid morphology (Fig. 7a). DEtail-seq signals were comparable among samples grown at 12, 17, 22, and even 37 °C (Fig. 7b and c). However, ptDNA was severely damaged at 44 °C after just 5 h (Fig. 7b). Unlike the ptDNA break pattern observed in the *atnh1c* mutant (Fig. 4a), the extensive breaks at 44 °C were more randomly distributed across the plastid genome, including the SSC region (Fig. 7b and Figure S10a). Compared to the break level at 37 °C, ptDNA break at 44 °C increased approximately 2-fold overall and in the SSC region (Fig. 7c and d). The ptDNA break pattern at 44 °C showed much lower correlation with those at other temperatures (Fig. 7e). In contrast, seedlings grown at 4 °C exhibited less than half the ptDNA break level of those grown at 22 °C (Fig. 7c).

Given that AtRNH1C plays a crucial role in DNA damage repair (Wang et al. 2021), we sought to determine whether the unaffected ptDNA break level under most tested temperatures is at least partially attributable to the capacity of DNA damage repair. To this end, we examined ptDNA integrity in *atrnh1c* under different temperatures (4, 22, and 37 °C). After 2 d of treatment at 37 °C, *atrnh1c* exhibited an aggravated yellowish phenotype (Fig. 7a), indicating heightened sensitivity to high temperatures. Compared to the break levels at 22 °C, DEtail-seq signals in *atrnh1c* increased approximately 2-fold at 37 °C (Fig. 7b and c). In contrast, ptDNA breaks in *atrnh1c* grown at 4 °C were reduced, although not as significantly as that observed in Col-0 (Fig. 7c and Figure S10a). In line with the above observations, the temperature variation tested had no obvious effect on the stability of the SSC region in *atrnh1c* (Fig. 7b and d, and Figure S10a).

**Figure 8:**
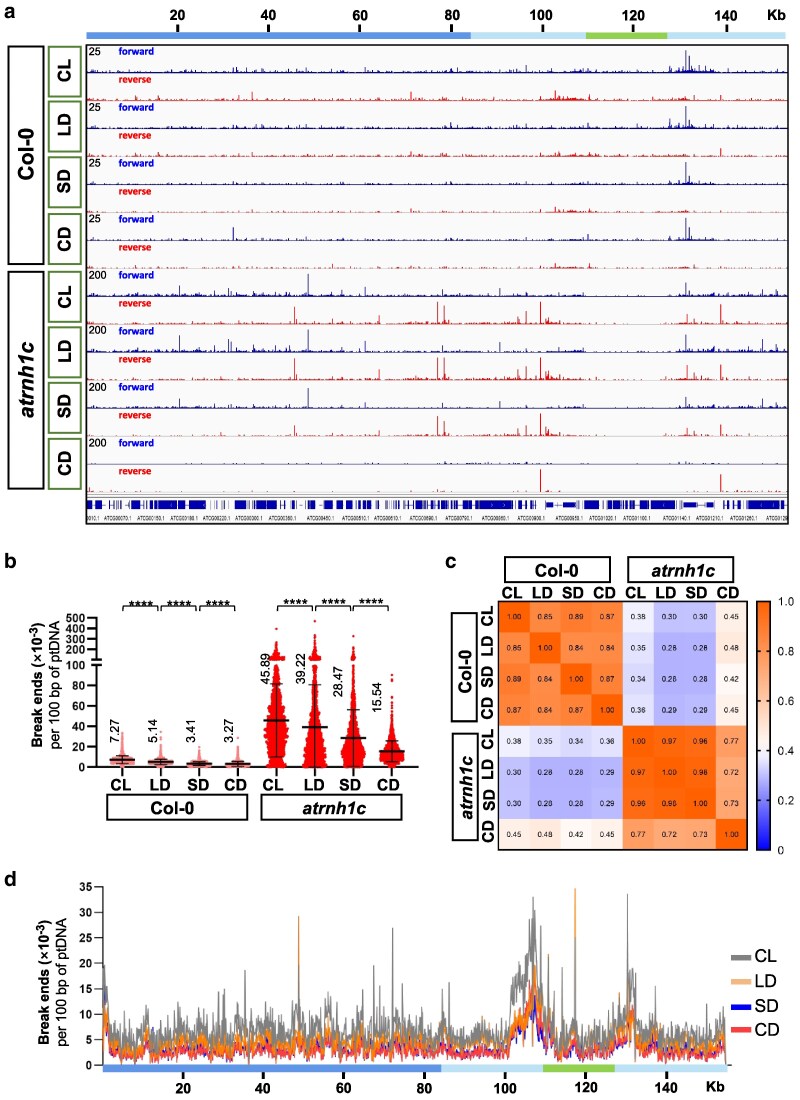
Shortening the photoperiod alleviates ptDNA damage. a) Overview of DNA break patterns in 16-day-old Col-0 and *atrnh1c* seedlings grown under different photoperiods for 5 d. b) Comparison of ptDNA break levels in different samples. Numbers adjacent to scatter points represent mean values. Central line: mean (values shown adjacent to scatter points). Error bars: SD. Asterisks: Wilcoxon matched-pairs signed rank test, ****P < 0.0001. c) Heatmap of Spearman's rank correlation coefficients between pairs of samples. d) Comparison of DNA break intensity on the reverse strand along the plastid genome in the samples indicated in (a). The signal was summed in consecutive, nonoverlapping 100-bp windows across the genome to generate the line plot. CL, constant light; LD, long day; SD, short day; CD, constant dark.

As photoperiod variation represents another common environmental stimulus, we sought to analyze its effect on plastid genome stability. Sixteen-day-old Col-0 seedlings grown under long-day (16 h light/8 h dark) conditions were transferred to growth chambers with different photoperiods: constant light (CL, 24 h light), long-day (LD, 16 h light/8 h dark), short-day (SD, 8 h light/16 h dark), and constant dark (CD, 24 h dark). They were grown for an additional 5 d. While the CD treatment caused seedling etiolation, the morphology of the chloroplast nucleoid remained largely unaffected (Figure S11a and b). Overall, DEtail-seq signals were similar in CL and LD samples but slightly reduced in SD and CD samples (Fig. 8a and b). We then investigated whether a shortened period could also alleviate ptDNA damage in the *atrnh1c* mutant. The same treatments were applied to 16-d-old *atrnh1c* seedlings. Indeed, DEtail-seq signals in *atrnh1c* decreased slightly as the photoperiod shortened (Fig. 8a and Figure S10b). The normalized ptDNA break level in the CD-treated sample was only one-third of that in CL-treated samples (Fig. 8b), despite the severe yellowish phenotype observed in seedlings after CD treatment (Figure S11a). Correlation analysis revealed that different light conditions do not significantly alter the patterns of ptDNA break (Fig. 8c). Consistently, a similar break intensity pattern was observed in consecutive, nonoverlapping 100-bp windows, although slight variation was observed in some small regions (Figure S11c).

## Discussion

In this study, we mapped genome-wide DNA breaks in plastids across different tissues and developmental stages by precisely locating their 3′ ends. DSBs are primarily repaired through 2 main pathways: homologous recombination (HR) and nonhomologous end joining (NHEJ) (Scully et al. 2019). NHEJ can be further categorized into classical nonhomologous end joining (c-NHEJ) and alternative nonhomologous end joining (alt-EJ), the latter also known as microhomology-mediated end joining (MMEJ) (Ceccaldi et al. 2016; Scully et al. 2019). In plastids, HR and MMEJ are identified as the predominant DSB repair pathways, while there is no evidence supporting the existence of c-NHEJ (Kazama et al. 2019; Chevigny et al. 2020). Both HR and MMEJ rely on the 3′ overhangs generated at DSB ends (Symington and Gautier 2011), making it reasonable to conclude that 3′ overhangs are a prominent feature of DSBs.

The maintenance vs degradation of ptDNA during seedling development and tissue maturation has long been a subject to debate (Kumar et al. 2014; Ma and Li, 2015; Greiner et al. 2020). In this study, we employed the DEtail-seq method to map and quantify 3′ break ends in the plastid genome, analyzing both the same tissue across various seedling ages and different tissues at the same developmental stage with high precision. Our results demonstrate that ptDNA breakage generally increases during seedling development (Figs 2 and 3).

We propose that this increase arises from the convergence of developmentally programmed shifts: First, as leaves mature, the photosynthetic apparatus reaches peak capacity, inherently elevating electron transport flux and the production of ROS (Figure S9c)—a direct cause of DNA damage (Zhong et al. 2019; Gendron and Staiger 2023; Shao et al. 2024). This trend aligns with reports of accumulating oxidative and glycation damage in plant orgDNA over time, which are precursors to strand breaks (Tripathi et al. 2020, 2022). Second, concurrent with this rising damage potential, we observed a developmental decline of the expression of key nuclear-encoded genes essential for ptDNA stability (eg, *WHY1*, *WHY3*, *RECA1*, and *RNH1C*; Figure S12), indicating a reduced repair and recombination capacity (Wang et al. 2021). Third, in later stages, resources are prioritized for nutrient recycling over maintenance, and the autodegradation of chloroplast components, including DNA, is a hallmark of senescence (Sakamoto and Takami 2018). Thus, the net accumulation of ptDNA breaks likely results from this interplay between increasing damage potential and diminishing repair capacity.

Interestingly, ptDNA breaks increased dramatically in freshly emerged true leaves at the 4-week stage (Fig. 2), a phase coinciding with bolting under our growth conditions (Fig. 2b and Figure S2a). This distinct pattern may serve a specific physiological function. Recent studies have shown that chloroplast defects and HL treatment induce early flowering through retrograde signaling (Feng et al. 2016; Susila et al. 2023). As the newly formed rosette leaves at this stage may play a critical role in reproduction, the increased ptDNA breaks within them might generate retrograde signals that promote the transition from vegetative to reproductive growth. Further research is needed to explore the relationship between chloroplast genome stability and plant reproduction. Additionally, we observed that ptDNA breaks in seeds are significantly lower than in other tissues (Fig. 3), which is consistent with the notion that chloroplasts play a limited role in embryo development.

A growing body of evidence now indicates that DNA damage is strongly correlated with transcription (Gaillard and Aguilera 2016). We previously determined that the IR regions are susceptible to breakage due to the high transcriptional activity of rDNAs and the presence of replication origins, which frequently leads to TRCs (Yang et al. 2020). Using DEtail-seq, we observed prominent break peaks in rDNA regions (Figures S3e and S4b), which aligned precisely with the enrichment of RpoB in these regions (Figs 2 and 3a, and Figure S3a). Furthermore, we found that breaks preferentially occur on the template strand within the rDNA regions (Fig. 2d, Figures S3e and S4b), suggesting that TRC-induced breaks predominantly arise on the transcriptional template strand. However, the loss of SIGMA6 had no significant effect on ptDNA stability (Fig. 6a and Figure S8a), likely because SIGMA6 is dispensable for 3-week-old seedlings. This may be attributed to functional redundancy among the 6 SIGMA factors in Arabidopsis plastids (Puthiyaveetil et al. 2021).

In chloroplasts, 2 types of RNA polymerase are active: the nuclear-encoded RNA polymerase (NEP) and the PEP, both of which function throughout all developmental stages. Therefore, the ptDNA breaks observed in RpoB-unbound regions (Figs 3a and 4a) may partially result from the activity of NEP. Overall, ptDNA breaks showed no clear preference for either gene regions or intergenic regions, nor for the template strand vs coding strand (Fig. 4c and Figure S6c). We propose that this lack of preference may stem from several features of plastid gene expression: (i) the transcription initiation and termination are loosely controlled (Castandet et al. 2019), (ii) numerous noncoding RNAs are frequently transcribed (Zhelyazkova et al. 2012), and (iii) the plastome is suggested to be fully transcribed (Shi et al. 2016).

**Figure 9:**
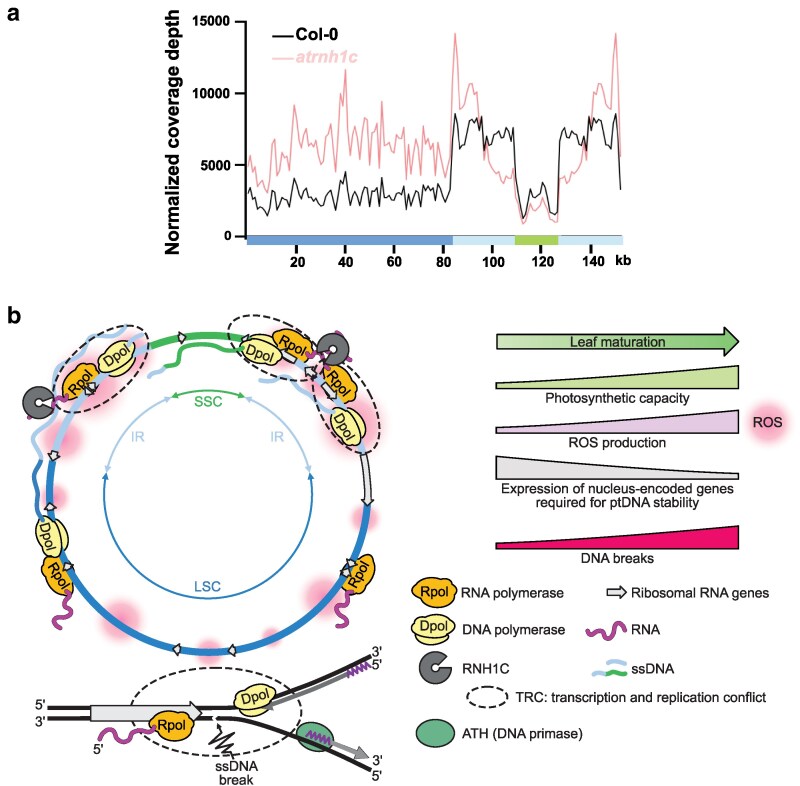
Model of TRC leading to DNA breaks along the plastid genome. a) Plastid sequencing coverage in 3-week-old Arabidopsis seedlings of Col-0 and *atrnh1c*. Data were retrieved from accession number PRJNA698745 (Wang et al. 2021). Positions were rounded down to 1 kb. Reads mapping to the plastid large inverted repeats (IRs) were equally distributed between the 2 IRs. The *y* axis represents the number of reads per million total plastid reads. b) In the plastid genome, TRCs occur frequently in the LSC and even more frequently in the IRs, but rarely in the SSC, posing a threat to genome stability in the IRs and LSC. AtRNH1C plays a role in resolving TRCs.

Compared to Col-0, the ptDNA break levels were significantly increased in *atrnh1c* and *reca1*/*atrnh1c* and further elevated in *why1*/*3*/*atrnh1c* across the entire genome, except for the SSC region, which remained relatively unaffected, forming a BSR (Fig. 4). Previous studies (Ret et al. 2018) and our recent deep-sequencing data (Wang et al. 2021) revealed that the DNA copy number is highest in the IR regions, but lowest in the SSC region (Fig. 9a), suggesting that DNA replication is highly active in the IRs but minimal in the SSC. Combined with the RpoB enrichment pattern, we propose that the BSR arises from extensive TRCs in IRs and LSC, and fewer TRCs in SSC (Fig. 9b).

Chloroplasts are widely regarded as the most optimal plant organelle for detecting environmental fluctuations (Crosatti et al. 2013; Kleine et al. 2021). Consistent with this role, chloroplasts were able to maintain genome stability under most conditions tested (Figs 6 and 7), aligning with the observation that the chloroplast genome is even more stable than the nuclear genome (Drouin et al. 2008). These findings suggest that plastids possess robust DNA damage repair and/or prevention mechanisms. In our recent work, we determined that AtRNH1C plays a critical role in DNA damage repair (Wang et al. 2021). Therefore, the exacerbated ptDNA damage in *atrnh1c* at 37 °C results from impairment of DNA damage repair. Interestingly, ptDNA damage was significantly reduced at 4 °C but not at 12 °C in Col-0 (Fig. 6b and c). Previous studies have shown that chloroplast transcription is disrupted at 4 °C, but remains largely unaltered at 12 °C (Wang et al. 2016; Gao et al. 2022). Thus, we speculate that the reduction in ptDNA damage at 4 °C may be due to the alleviation of TRCs. This hypothesis is further supported by the observation that DNA breaks in the *atrnh1c* mutant are also rescued under 4 °C (Fig. 7).

Recently, Tripathi and colleagues demonstrated that glycation damage and oxidative damage in maize ptDNA were significantly lower in plants grown in the dark compared to those grown under light (Tripathi et al. 2020, 2022). Therefore, we were not surprised to observe a reduction in ptDNA breakage with shortened photoperiod in Col-0. However, it was unexpected that this reduction was even more pronounced in the *atrnh1c* mutant (Fig. 7a and b). As the center of photosynthesis, chloroplasts generate ROS through photosynthetic electron transfer, a process that is highly dependent on photoperiod, light intensity, and quality (Li and Kim 2022). Mounting evidence suggests that ROS can trigger R-loop accumulation and genome instability (Teng et al. 2018; Tan et al. 2020; Renaudin et al. 2021). Exploring the relationship between R-loops and ROS in plastid genome stability represents an intriguing avenue for future research.

Plastids represent a diverse group of organelles, including chloroplasts, amyloplasts, chromoplasts, dessicoplasts, elaioplasts, etioplasts, gerontoplasts, leucoplasts, phenyloplasts, proplastids, and proteinoplasts, to only cite a few, with chloroplasts being the predominant type in most tissues. These organelles vary in size, shape, and function and are distributed across different cell types (Pinard and Mizrachi 2018). The plastids isolated in this study were at the organ scale, representing a mixture of plastids from multiple cell types. Consequently, the results reflect an average across these diverse plastid populations. To achieve a higher resolution of ptDNA break patterns, a method to isolate plastids from specific cell types may be required. Recently, Boussardon et al. developed a technique, called IPTACT (Isolation of Plastids TAgged in Specific Cell Types), which was used to isolate plastids from mesophyll and companion cells of *A. thaliana* (Boussardon et al. 2023). Combining DEtail-seq with IPTACT could provide deeper insights into ptDNA break patterns at the cellular level.

## Materials and methods

### Plant materials and growth conditions

All *A. thaliana* plants used in this study were of the Columbia-0 (Col-0) accession. The mutants *atrnh1c*, *reca1*/*atrnh1c*, *why1*/*3*/*atrnh1c*, *reca1*, *why1*/*3*, *polIa*, *polIb*, *why1*/*3*/*reca1*, *why1*/*3*/*polIb* have been described previously (Wang et al. 2021; Zhang et al. 2024). Seeds were surface-sterilized and sown on Petri plates containing 1/2 Murashige and Skoog (MS) medium (Murashige and Skoog salt base (Sigma-Aldrich), 0.8% [w/v] Phytagr (ThermoFisher Scientific), and 1% [w/v] Sucrose). The plates were stored in the dark at 4 °C for 2 d before being transferred to a growth chamber. After 1 wk of cultivation, seedlings were transplanted onto fresh 1/2 MS medium or soil for further growth. Both plate-grown and soil-grown plants were maintained in growth chambers under long-day conditions (16 h light/8 h dark, 22 °C/20 °C) with an average photon flux density of 120 to 150 μE m^−2^ s^−1^. Seedlings were counted from the day the plates were moved into the growth chamber.

For tissues at different development stages, seeds were sown simultaneously, and tissues were collected weekly as indicated for chloroplast isolation.

For temperature treatments, seedlings were grown on plates for 19 d, then transferred to growth chambers and exposed to different temperatures under long-day conditions for 2 d. Aerial parts were harvested for chloroplast isolation.

For chemical treatments, 16-d-old seedlings were transferred onto fresh 1/2 MS medium supplemented with either 0.5 mM AsA or 2 mM H_2_O_2_, and then grown under the specified light conditions for 5 d before sampling.

For light intensity and quality treatment, seedlings were grown on plates for 16 d, then transferred into growth chambers and exposed to different light intensities/qualities (strong light 180 μE m^−2^ s^−1^, weak light 60 μE m^−2^ s^−1^, blue light 120 μE m^−2^ s^−1^, red light 120 μE m^−2^ s^−1^) for 5 d. Aerial parts were harvested for chloroplast isolation.

For photoperiod treatment, seedlings were grown on plates for 16 d, then transferred into growth chambers and exposed to different photoperiods for 5 d. Aerial parts were harvested for chloroplast isolation.

### Chloroplast isolation

Chloroplast isolation was performed as previously described (Wang et al. 2021). Briefly, the plant material was harvested and ground in ice-cold chloroplast isolation buffer (CIB, 10 mM HEPES–KOH [pH 8.0], 150 mM sorbitol, 2.5 mM EDTA [pH 8.0], 2.5 mM EGTA [pH 8.0], 2.5 mM MgCl_2_, 5 mM NaHCO_3_, and 0.1% BSA). The homogenate was filtered through 3 layers of Miracloth (Millipore) and centrifuged at 200 × *g* at 4 °C for 3 min to remove whole cells and cell wall debris. The supernatant was then transferred to fresh, prechilled 50-mL tubes and centrifuged at 1,000 × *g* at 4 °C for 10 min. The resulting chloroplast pellet was used immediately for the subsequent steps.

### DEtail-seq

The chloroplasts were embedded in 0.5% low-melting-point agarose (Promega) and lysed in a solution containing 1% sarkosyl, 0.45 M EDTA (pH 8.0), 10 mM Tris–HCl (pH 8.0), and 2 mg/mL proteinase K at 48 °C for 16 h with shaking. The agarose plugs were washed 6 times in TE buffer at 37 °C, with the first 2 washes supplemented with 1 mM PMSF. The plugs were cut into 12 pieces and incubated in 1 mL TE buffer containing 10 μg/mL RNase A at 37 °C overnight. After incubation, the agarose pieces were washed 5 times with 1 mL TE buffer at 37 °C for 6 min each and stored in plug storage solution (0.05 M EDTA [pH 8.0], 60% glycerol) at −20 °C until all samples were ready for the next step.

The DNA library was constructed using the Scale ssDNA-seq Lib Prep Kit for Illumina V2 (ABclonal) following a previously described protocol with modifications (Xu et al. 2023). Briefly, 8 agarose pieces (∼50 mg) were washed 3 times with 200 μL 1× CutSmart buffer (NEB) at 37 °C for 6 min each. Spike-in digestion was performed by incubating the agarose pieces with 15 U *I-CeuI* (NEB) in 150 μL 1× CutSmart buffer at 37 °C for 12 h. These pieces were later used to purify DNA (using Magen kit, Cat. No. D2111-02) to determine the cleavage efficiency via qPCR with specific primers (Table S2). The remaining 5 agarose pieces were processed for library construction. They were washed 3 times with 500 μL TE buffer at 37 °C for 6 min, followed by 2 additional washes with 200 μL Low EDTA TE (ABclonal) at 37 °C for 6 min. The agarose pieces were then incubated in 80 μL T7 Tailing & Ligation buffer (ABclonal) at 37 °C for 12 h to ligate the ssDNA adapter to the 3′ ends of the DNA break sites. Subsequently, the agarose pieces were heated at 98 °C for 2 min. DNA was recovered from the agarose using a DNA purification kit and eluted with Low EDTA TE. The DNA was sonicated to ∼250 bp using the Focused-ultrasonicator (Covaris S220). The remaining steps of library preparation were performed according to the manufacturer's protocol. Finally, the libraries were sequenced on the Illumina NovaSeq 6000 platform (150 bp pair-end reads).

### ssDRIP-seq

The extraction of ptDNA and the construction of the ssDRIP-seq library were performed as previously described (Yang et al. 2017; Xu et al. 2020). Briefly, 2 μg of ptDNA was fragmented using 1 U each of DdeI, MseI, RsaI, and AluI (New England Biolabs) at 37 °C for 12 h, followed by purification using the phenol–chloroform method. Next, 1 μg of fragmented DNA was incubated with 10 μg of S9.6 antibody at 4 °C overnight, followed by further incubation with 50 μL Protein G beads (Invitrogen) for 4 h. The DRIPed DNA was purified using phenol–chloroform and sonicated to ∼250 bp. Library construction was then performed using the Accel-NGS 1S Plus DNA Library Kit (Swift Biosciences). The libraries were quality-checked on an Agilent BioAnalyzer and subsequently sequenced on an Illumina HiSeq X Ten system.

### Sequencing data processing

For ssDRIP-seq data, reads were aligned to the TAIR10 genome using Bowtie 2 with default settings. Duplicate reads were removed using Picard tools (http://broadinstitute.github.io/picard). The total mapped reads were separated into forward and reverse strands using samtools. For visualization, the aligned reads (Binary Alignment Map [BAM] files) were converted to normalized coverage files (bigWig) with 1-bp bins using bamCoverage from deepTools. Snapshots of the data were generated using the Integrative Genomics Viewer.

The DEtail-seq data were processed as previously described (Xu et al. 2023). First, duplicated reads with identical forward and reverse sequences were removed using a custom script (https://github.com/PEHGP/DEtail-seq/blob/master/RemoveSamReads.py). The remaining reads were aligned to the reference genome using Bowtie2 with the –local setting. The reads mapped to 2 inverted repeats were randomly distributed to IR1 or IR2. For visualization and splitting strands, the aligned reads (BAM files), including the second read in pairs, were converted to single-base bigWig files with 1 bp bins using bamCoverage from deepTools with the following parameters: –binSize 1, –Offset 1, –samFlagInclude 128. Due to the single-stranded nature of the starting DNA in the scale ssDNA-seq library, the strand specificity of the sequenced reads is inverted relative to standard double-stranded libraries. Accordingly, to generate bigWig files for the reverse strand, we used –filterRNAstrand forward; for the forward strand, we used –filterRNAstrand reverse. Normalization was performed using deepTools based on CPM (Counts Per Million mapped reads).

### Identification of DNA breaks and normalization

To identify genomic positions corresponding to significant DNA strand breaks (hotspots), we performed statistical peak calling on the CPM-normalized bigWig files using a custom Python script (https://github.com/PEHGP/DEtail-seq/blob/master/Hotspotcalling.py). For cross-sample comparability, an internal scaling factor was applied. First, for each sample, we calculated the average CPM-normalized signal across all *I-CeuI* recognition sites (±8 bp) and used this value as a sample-specific internal scaling factor (Scale). This approach is valid because digestion efficiency at these sites was verified to exceed 99.5%, effectively representing a complete cut and thus a constant, sample-specific background. All CPM values were then divided by this Scale to produce the final normalized signal track. Therefore, the break values reported throughout the manuscript represent relative break signal intensity, normalized to the sample-specific *I-CeuI* background, and are directly comparable between samples. For enhanced visualization in IGV and downstream analysis, the normalized values were subsequently multiplied by 10^4^.

Break hotspots were identified from the CPM-normalized data through statistical testing. A genome-wide background signal intensity (mean) was calculated. Each base position with a positive signal was tested against this background using a Poisson model to compute a *P*-value. *P*-values were adjusted genome-wide using the Benjamini–Hochberg false discovery rate (FDR) correction. A position was definitively called as a DNA break hotspot only if its FDR *q*-value was ≤0.1. Signals within I-CeuI site regions were excluded from all final analyses and visualizations.

### R-loop and DNA break analysis

To perform a genome-wide analysis of R-loop and DNA break signals in the plastid, we employed a window-based approach. The chloroplast genome is divided into nonoverlapping 100-bp windows. For each window, we calculated signal intensities (Mean R-loop [ssDRIP-seq] and DNA break [DEtail-seq] signals for both Col0 and *atrnh1c*) and fold changes (FC): log_2_-transformed FC for both signals between genotypes (log_2_FC = log_2_[(*atrnh1c* signal + *ε*)/(Col-0 signal + *ε*)], where *ε* = 1 × 10^−10^). The concordance between the log_2_FC values of R-loop and DNA break signals across all windows was evaluated using Spearman's rank analysis. Statistical significance was determined using 2-tailed tests with *α* = 0.05.

### Pulsed-field gel electrophoresis

The PFGE assay was performed as previously described (Wang et al. 2021). Chloroplasts were embedded in 0.5% low-melting-point agarose and lysed in a solution containing 1% sarkosyl, 0.45 M EDTA, 10 mM Tris–HCl (pH 8.0), and 2 mg/mL proteinase K at 48 °C for 16 h with shaking. Agarose plugs were washed 6 times in TE buffer at 4 °C, with the first 2 washes supplemented with 1 mM PMSF. The plugs were then loaded into a 1% agarose gel. Electrophoresis was performed using a CHEF Mapper XA system (BioRad) with switch times ramped from 5 to 120 s at 4.5 V/cm. After staining with EtBr and imaging, the gel was blotted onto a Hybond N^+^ membrane (GE Healthcare Life Science). A 505-bp fragment of the chloroplast *rbcL* gene (55,677 to 56,181) was labeled with [α-^32^P]dCTP using the Random Primer DNA Labeling Kit Ver. 2 (Takara) and used as a probe for hybridization. The autoradiograph was exposed to phosphor screens for 3 d and scanned using a Typhoon FLA9500 scanner.

### DAPI staining and microscopy

Leaf tissues were cut into sections of approximately 0.5 × 0.5 cm^2^ and fixed in 3.5% (v/v) glutaraldehyde solution prepared in ddH_2_O for 1 h at room temperature under dark conditions. After 2 washes with ddH_2_O, the tissues were incubated in EDTA-Na at 60 °C for 3 h, followed by overnight incubation at 4 °C. The tissues were then placed on glass slides and covered with mounting medium (SouthernBiotech) containing 4′,6-diamidino2-phenylindole (DAPI). Cover glasses were mounted, and samples were observed using a confocal laser-scanning microscope (Zeiss LSM880 or Leica SP8).

### Reactive oxygen species detection assays

For the subcellular detection of reactive oxygen species (ROS) in Arabidopsis leaves, seedlings were submerged in 15 µM H_2_DCFDA (ApexBio, cat. C3890) solution and incubated for 15 min in the dark. Following incubation, samples were rinsed 3 times with 1× PBS buffer over a period of 15 min. Imaging was performed using a confocal laser-scanning microscope (Leica SP8).

For in situ detection of superoxide anion (O_2_^−^), seedlings were immersed in 1 mg/mL nitroblue tetrazolium (Sigma, Cat. No. N6876) staining solution prepared in 1× PBS and subjected to vacuum infiltration for 1.5 h. Subsequently, decolorization was conducted overnight using absolute ethanol. The samples were then transferred to a solution containing 25% glycerol and 75% ethanol for 15 min before being photographed with a digital camera.

### 8-oxoG detection assay

The purified chloroplasts were resuspended in 8% paraformaldehyde. A volume of 10 µL of the suspension was spotted onto an adhesive-coated slide and fixed for 30 min. After air-drying, the samples were post-fixed with 100 µL of 4% paraformaldehyde and air-dried again. The slide was then washed 3 times with 1× PBS, 2 min per wash. Next, the sample was covered with 1× PBS buffer containing 1% BSA and 0.15% Triton X-100, and incubated at room temperature for 20 min.

The primary Anti-8-oxoG antibody (Sigma-Aldrich, Cat. No. MAB3560) was diluted 1:100 in 1× PBS with 1% BSA and 0.15% Triton X-100. A total of 100 µL of the diluted antibody solution was applied to the slide and incubated overnight at 4 °C. The slide was then washed 3 times with 1× PBS for 2 min each. The fluorescent-conjugated secondary antibody, corresponding to the primary antibody, was diluted 1:100, applied to the slide (100 µL), and incubated for 1 h at room temperature. After 3 additional washes with 1× PBS (2 min each), 10 µL of DAPI-containing mounting medium was applied. A coverslip was carefully placed over the sample, and imaging was performed using a confocal laser-scanning microscope (Leica SP8).

### Slot-blot assay

Slot-blot assays were performed as described previously (Wang et al. 2021). Chloroplast DNA was treated with RNase III (1 U/5 µg DNA; NEB) at 37 °C for 2 h and purified using a HiPure Gel Pure DNA Mini Kit (Magen). A portion of the DNA was further digested with RNase H (1 U/µg DNA; NEB) at 37 °C for 1 h and repurified. Indicated amounts of DNA were blotted onto a Hybond N^+^ membrane (GE Healthcare). After UV crosslinking (1,200 µJ), total DNA loading was visualized by DuRed staining (Yeasen). The membrane was then probed with S9.6 antibody (1 µg/mL; from hybridoma HB-8730, ATCC) for R-loop detection.

### Accession numbers

*WHY1* (AT1G14410), *WHY3* (AT2G02740), *RecA1* (AT1G79050), *POLIA* (AT1G50840), *POLIB* (AT3G20540), *AtRNH1C* (AT1G24090), *SIGMA6* (AT2G36990), and *sigma6* (SAIL-645-F03).

## Supplementary Material

koag186_Supplementary_Data

## Data Availability

High-throughput sequencing data have been deposited in the Gene Expression Omnibus database (accession nos. GSE242392, GSE242391, GSE242392, GSE308159, GSE308160, GSE308349, and GSE317463).
