# Supplementary material for: Landscape of plastid DNA breaks in Arabidopsis during development and environmental stimulus
Source: Plant Cell. 2026 Jun 16;38(7):koag186. doi: 10.1093/plcell/koag186 (PMC13412044; doi:10.1093/plcell/koag186)
Supplement: koag186_Supplementary_Data [file koag186_supplementary_data.zip › TPC-2025-1025R1_Supplemental Data.pdf]

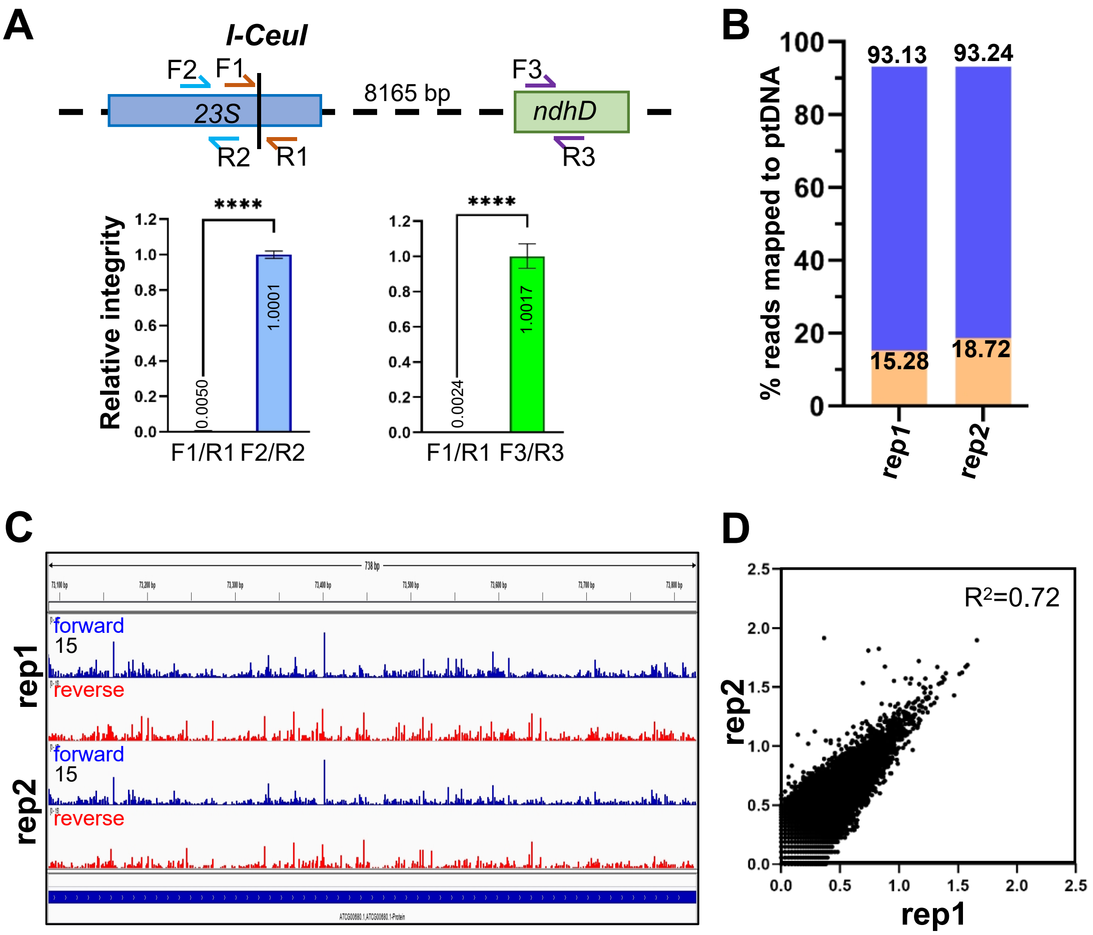

Figure S1. Validation of DEtail-Seq using 2-week-old seedlings.

(A) Determination of *in situ I-CeuI* digestion efficiency. Upper panel: schematic illustrating the locations of the *I-CeuI* cutting site and primer sets. Lower panel: qPCR analysis showing relative integrity at the *I-CeuI* cutting site, compared to an adjacent region (left) and a distal region (right). Error bars represent standard deviation (SD). Asterisks indicate statistically significant differences (Student's t-test, ****P < 0.0001). **(B)** Proportion of sequencing reads mapped to the plastid genome. The brown segment of each bar represents the subset of reads aligned specifically to the *I-CeuI* cleavage sites. **(C)** Another representative snapshot of a region demonstrating the similarity between two biological replicates. **(D)** Correlation of DEtail-seq signals between two biological replicates on the reverse strand. Normalized read counts ($\log_{10}(n+1)$) were compared at every single base, and Pearson's correlation coefficient (R^2) is indicated.

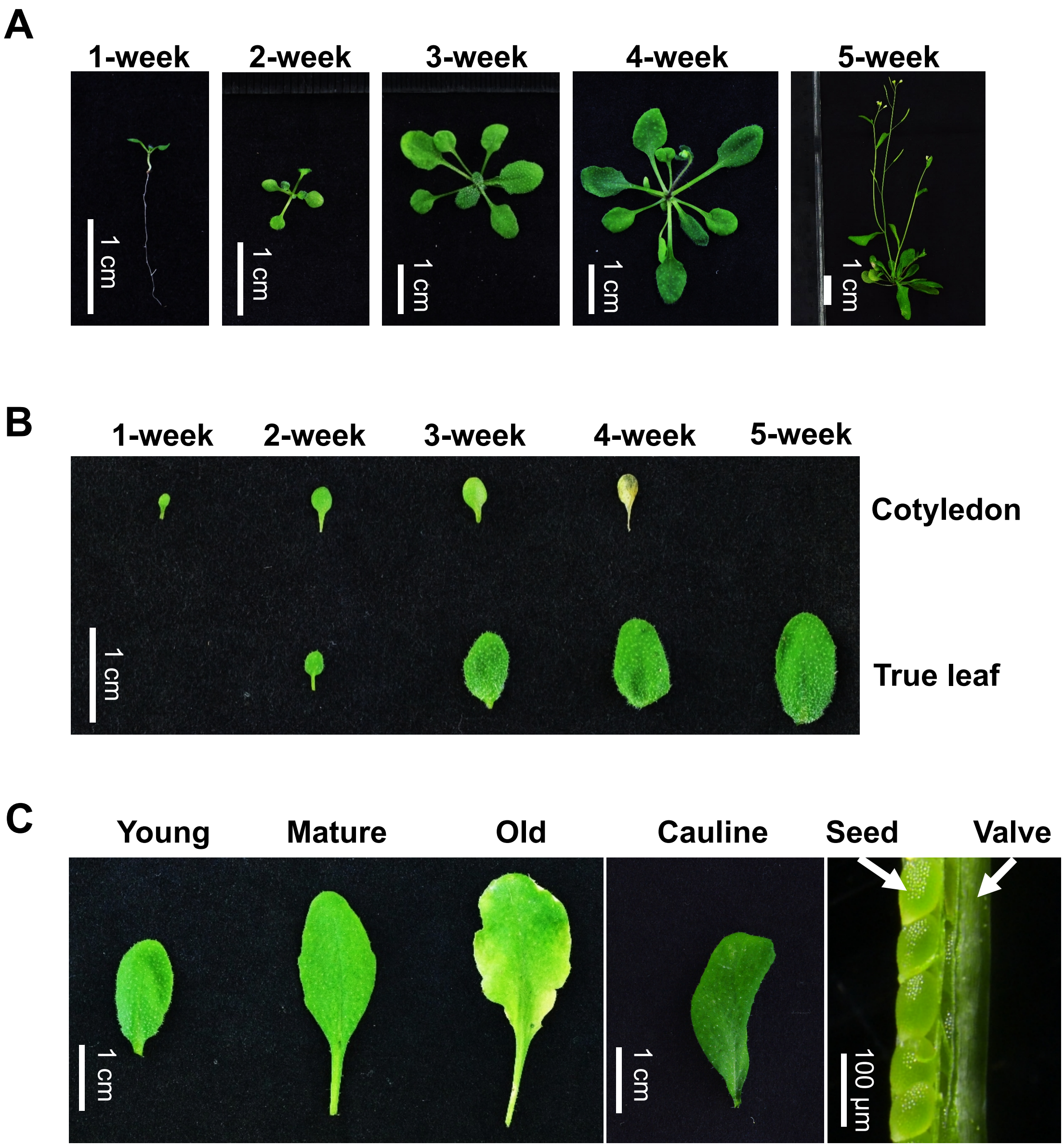

Figure S2. Seedlings and tissues used for DEtail-seq

(A) Images of 1- to 5-week-old Col-0 plants. **(B)** Images of cotyledons and freshly emerged true leaves from plants at the indicated ages. Scale bar: 1 cm. **(C)** Different tissues collected from 5-week-old plants.

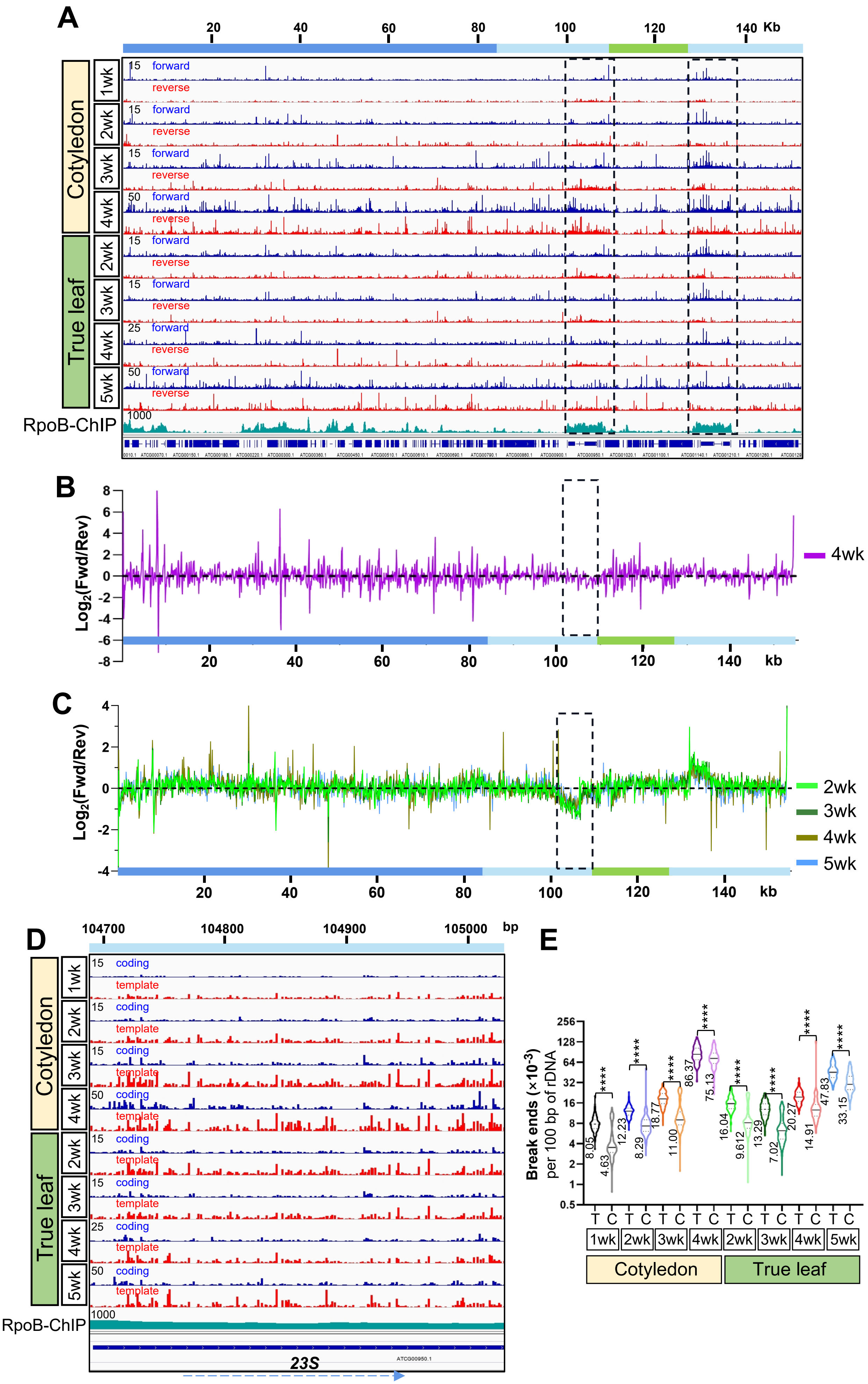
A
20
40
60
80
100
120
140 Kb
Cotyledon
True leaf
1wk
2wk
3wk
4wk
5wk
15
25
50
forward
reverse
RpoB-ChIP
1000
B
Log2(Fwd/Rev)
4wk
20
40
60
80
100
120
140
kb
C
Log2(Fwd/Rev)
2wk
3wk
4wk
5wk
D
104700
104800
104900
105000
bp
coding
template
23S
ATCG00950.1
E
Break ends (×10-3) per 100 bp of rDNA
256
128
64
32
16
8
4
2
1
0.5
****
8.05
4.63
12.23
8.29
18.77
11.00
86.37
75.13
16.04
9.612
13.29
7.02
20.27
14.91
47.83
33.15
T
C
1wk
2wk
3wk
4wk
2wk
3wk
4wk
5wk
Cotyledon
True leaf

Figure S3. ptDNA break symmetry between the two strands.

(A) An IGV snapshot of ptDNA break patterns using a smaller data range as indicated, to highlight the signals. The top track: genome coordinate (kb). The middle track: break ends as colored peaks (blue = forward strand, red = reverse strand); peak height indicates break frequency. The bottom track: annotated genes (green arrows) with transcription direction. **(B)** Comparison of break intensity between two DNA strands in cotyledons from 4-week-old seedlings. The ratio of break ends per 100 bp on the forward strand to that on the reverse strand is shown on a $\log_2$ scale. **(C)** Comparison of break intensity between two DNA strands in freshly emerging true leaves from 2-, 3-, 4-, and 5-week-old seedlings. The ratio of break ends per 100 bp on the forward strand to that on the reverse strand is shown on a $\log_2$ scale. **(D)** Enlarged view of a representative region in rDNA demonstrating asymmetric breaks between the coding and template strands. Blue arrowheads denote transcriptional direction. **(E)** Break intensity in coding and template strands in the rDNA region. Numbers adjacent to scatter points represent mean values. The central line indicates the median, and the lower and upper lines represent the first and third quartiles, respectively. Asterisks indicate statistically significant differences (Wilcoxon matched-pairs signed rank test, ****$P < 0.0001$). T: template strand, C: coding strand. The black-dotted box outlines the rDNA region. Fwd: forward, Rev: reverse.

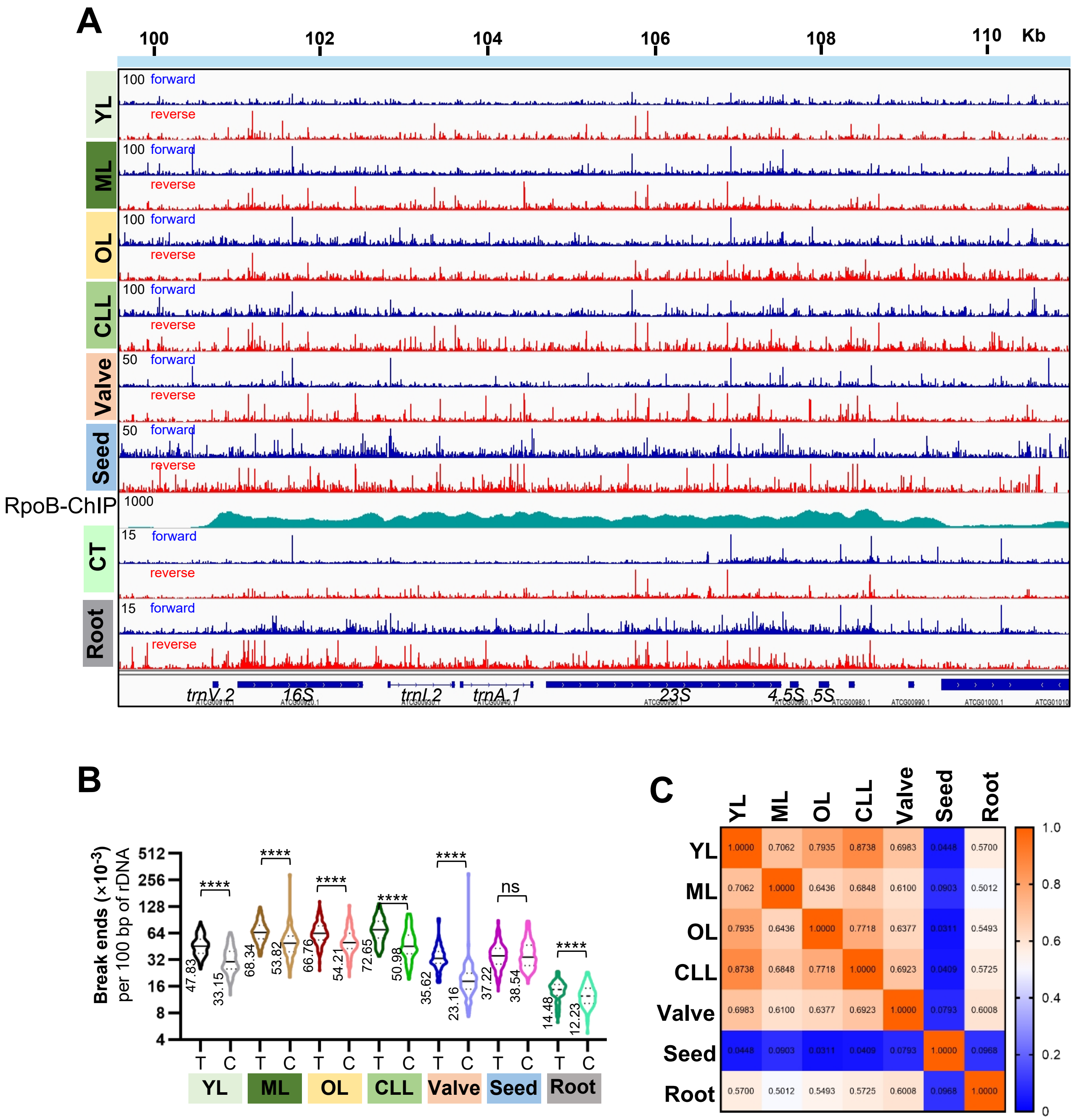

	YL	ML	OL	CLL	Valve	Seed	Root
YL	1.0000	0.7062	0.7935	0.8738	0.6983	0.0448	0.5700
ML	0.7062	1.0000	0.6436	0.6848	0.6100	0.0903	0.5012
OL	0.7935	0.6436	1.0000	0.7718	0.6377	0.0311	0.5493
CLL	0.8738	0.6848	0.7718	1.0000	0.6923	0.0409	0.5725
Valve	0.6983	0.6100	0.6377	0.6923	1.0000	0.0793	0.6008
Seed	0.0448	0.0903	0.0311	0.0409	0.0793	1.0000	0.0968
Root	0.5700	0.5012	0.5493	0.5725	0.6008	0.0968	1.0000

Figure S4. ptDNA break patterns in different tissues.

(A) Snapshot of the rDNA region in IR1. **(B)** Break intensity in coding and template strands in the rDNA region. Numbers adjacent to scatter points represent mean values. The central line indicates the median, and the lower and upper lines represent the first and third quartiles, respectively. Asterisks indicate statistically significant differences (Wilcoxon matched-pairs signed rank test, ****P < 0.0001). T: template strand, C: coding strand. **(C)** Heatmap of Spearman's rank correlation coefficients between pairs of samples.

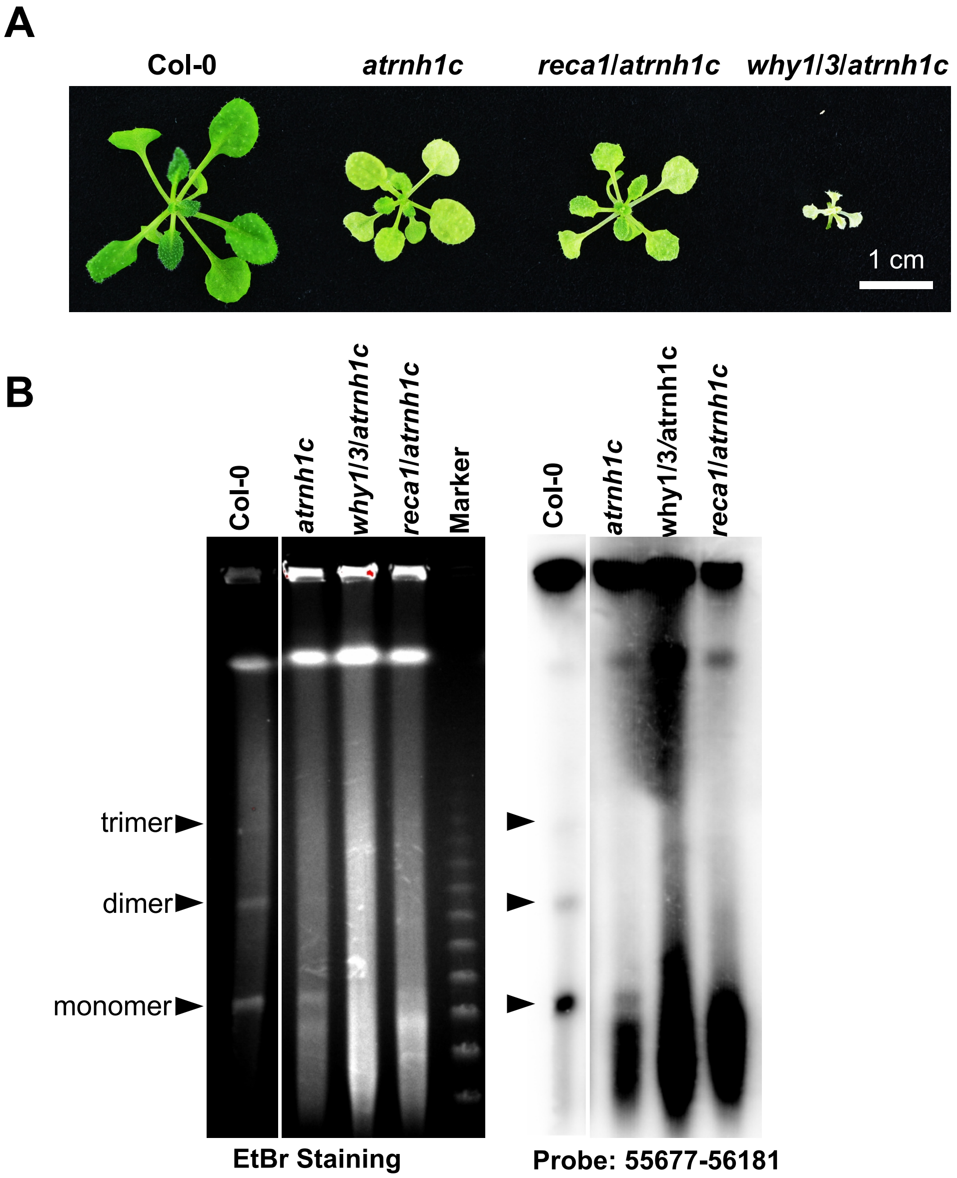

Figure S5. Phenotype and genome integrity of Col-0 and *atrnh1c* mutant.

(A) Phenotypes of 3-week-old seedlings of Col-0, *atrnh1c*, *reca1/atrnh1c*, and *why1/3/atrnh1c*. Scale bars: 1 cm. **(B)** PFGE assay of ptDNA from the seedlings described in **(A)**. The left panel shows the ethidium bromide-stained gel image. The right panel displays the blot hybridization using a 505-bp rbcL gene fragment (positions 55677–56181) as a probe. Arrowheads indicate bands corresponding to monomer, dimer, and trimer ptDNA molecules.

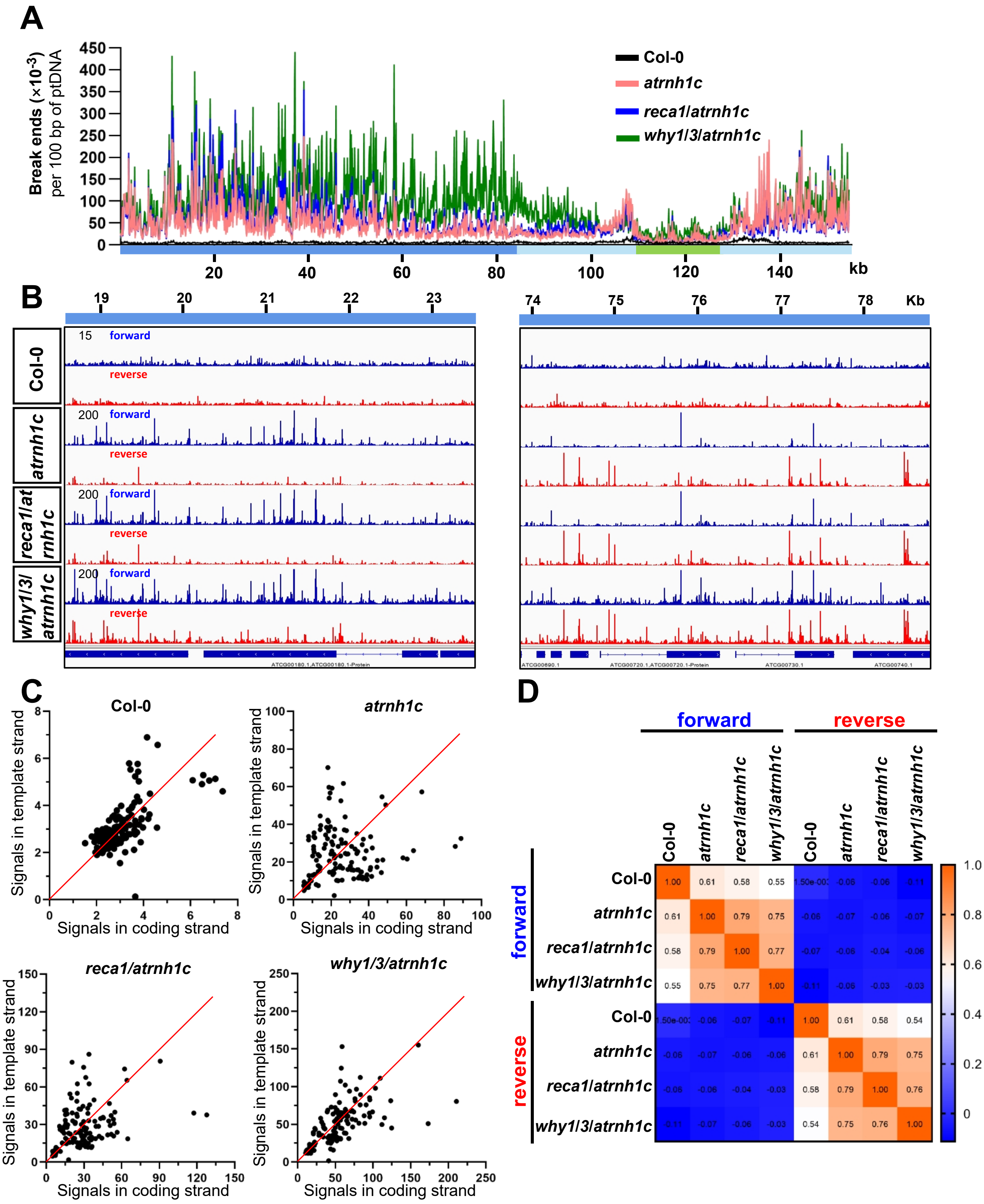

Figure S6. ptDNA breaks in different mutants.

(A) Comparison of DNA break intensity on the forward strand along the plastid genome in Col-0, *atrnh1c*, *reca1/atrnh1c*, and *why1/3/atrnh1c*. The signal was summed in consecutive, non-overlapping 100-bp windows across the genome to generate the line plot. **(B)** Two enlarged views of representative regions showing asymmetric break patterns. **(C)** ptDNA breaks show no clear preference for template strands or coding strands overall. Break intensity was compared between template strands and coding strands. **(D)** Heatmap of Spearman's rank correlation coefficients of forward and reverse strands between pairs of samples.

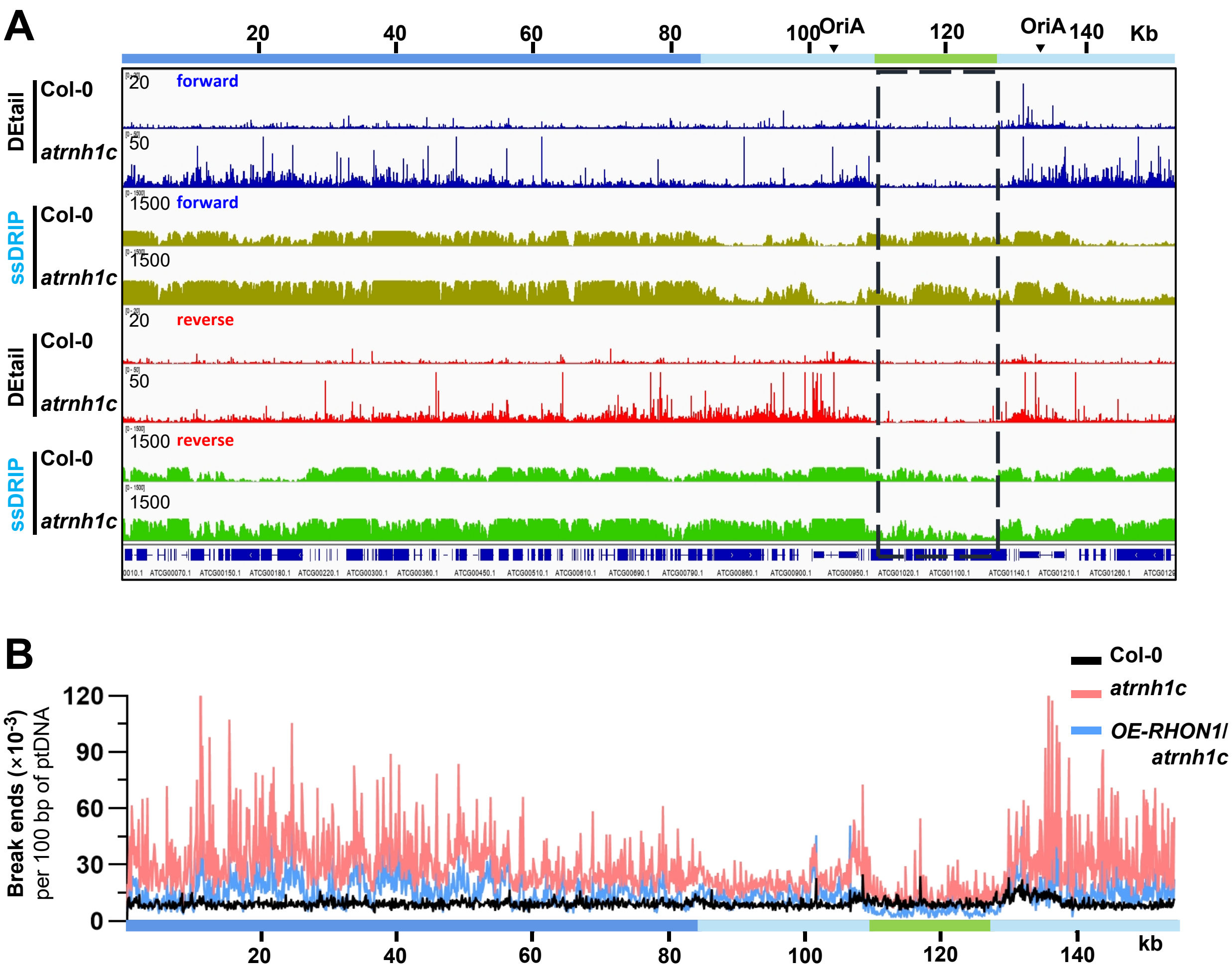

Figure S7. Profiling of DNA breaks and R-loop accumulation

(A) Genome-wide distribution of DEtail-seq (DNA break) and ssDRIP-seq (R-loop) signals across the plastid genome. **(B)** Comparison of forward strand ptDNA break intensity Col-0, *atrnh1c*, *reca1/atrnh1c*, and *why1/3/atrnh1c*. The signal was summed in consecutive, non-overlapping 100-bp windows to generate the line plot.

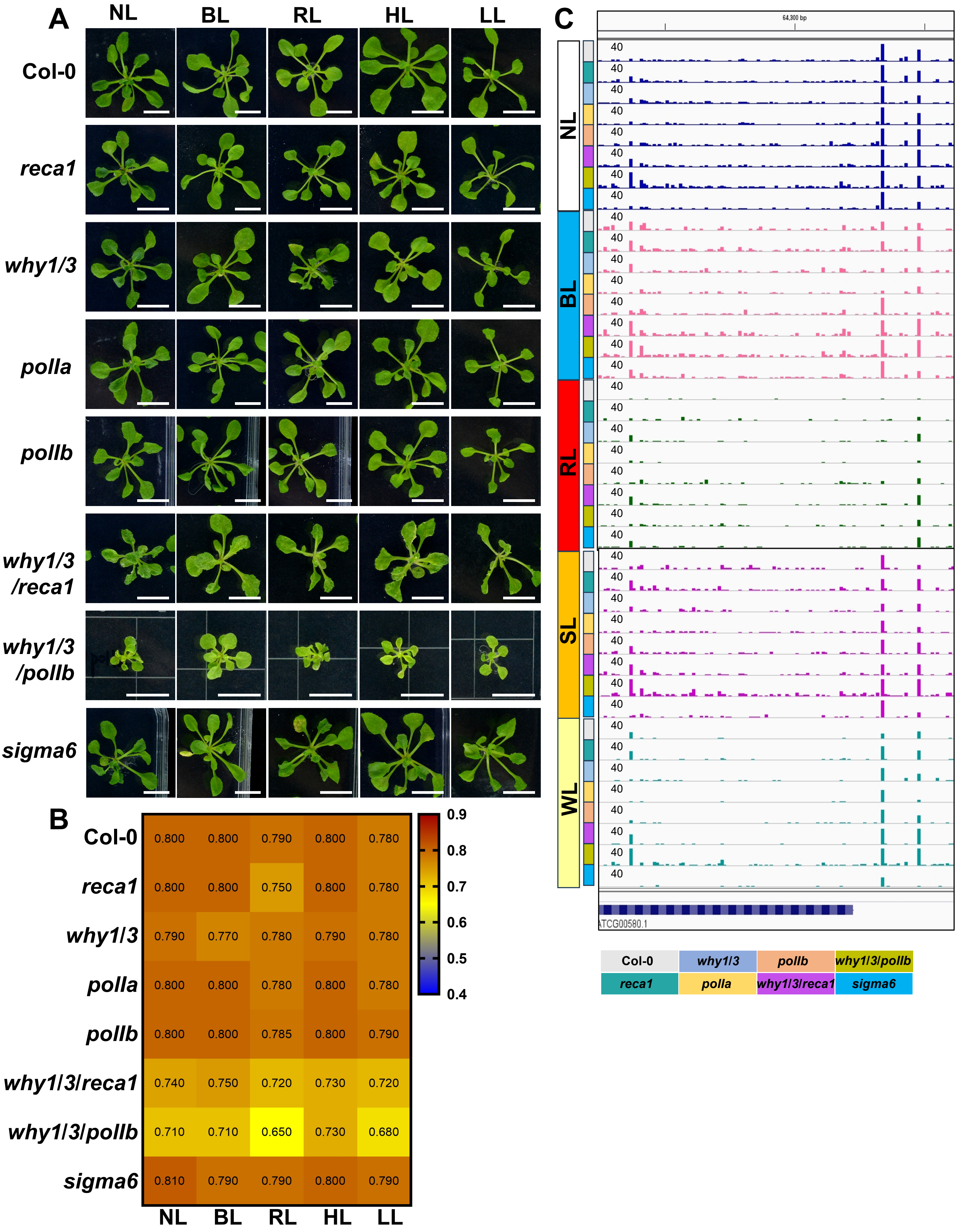

Figure S8. Growth phenotypes of mutants and DEtail-seq signals.

(A) Phenotype of mutants grown under different light qualities and intensities. **(B)** Representative IGV snapshots of DEtail-seq signals from all samples. **(C)** Chlorophyll Fv/Fm values of the plants shown in **(A)**. Scale bar: 1 cm. NL, normal light; BL, blue light; RL, red light; HL, high light; LL, low

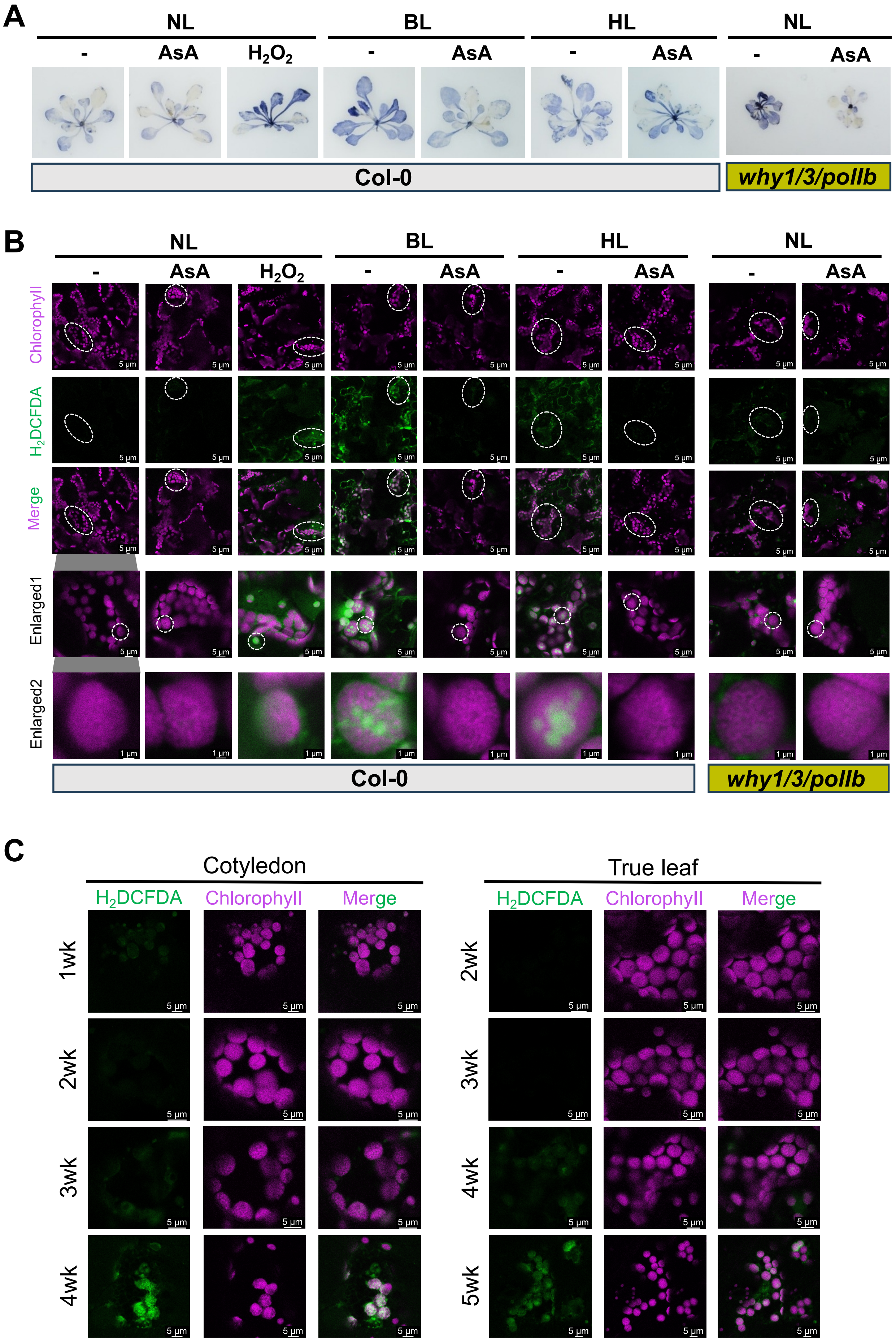
A
NL
BL
HL
NL
-
AsA
H2O2
-
AsA
-
AsA
-
AsA
Col-0
why1/3/pollb
B
NL
BL
HL
NL
-
AsA
H2O2
-
AsA
-
AsA
-
AsA
Chlorophyll
H2DCFDA
Merge
Enlarged1
Enlarged2
5 μm
1 μm
Col-0
why1/3/pollb
C
Cotyledon
True leaf
H2DCFDA
Chlorophyll
Merge
1wk
2wk
3wk
4wk
2wk
3wk
4wk
5wk
5 μm

Figure S9. The ROS accumulation under different treatments.

(A) NBT staining of seedlings indicating O_2^- levels. Sixteen-day-old seedlings grown on 1/2 MS medium under normal light were transferred to fresh 1/2 MS medium supplemented with 0.5 mM AsA or 2 mM H_2O_2 and cultured for an additional 5 days under the indicated light conditions. Whole seedlings were subjected to NBT staining. Blue coloration indicates O^{2-} accumulation. **(B)** H_2DCFDA fluorescence (indicative of ROS) in mesophyll cells from leaves of seedlings treated as in (A). Green signal represents oxidized H_2DCFDA, while red shows chlorophyll autofluorescence. White circles highlight enlarged regions of cells or chloroplasts. **(C)** H_2DCFDA fluorescence (indicative of ROS) in chloroplasts from cotyledons and true leaves of seedlings at 1, 2, 3, 4, and 5 weeks of age.

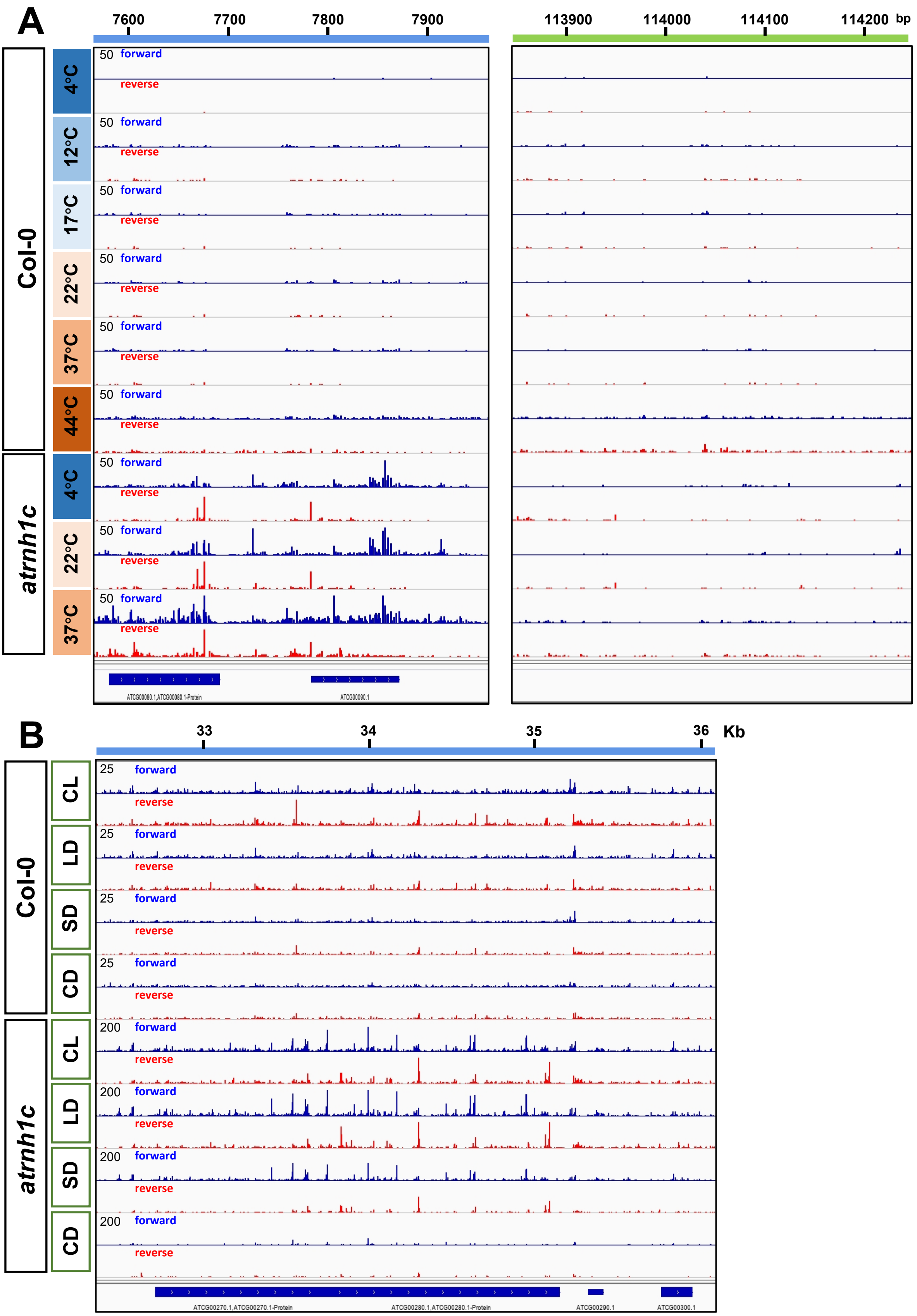

Figure S10. Enlarged view of DEtail-seq signals in Col-0 and *atrnh1c* in response to different environmental stimuli.

(A) Related to Figure 6A. Representative snapshots of DEtail-seq signals showing ptDNA break patterns in the LSC (left panel) and SSC (right panel) in Col-0 and *atrnh1c* under varying temperatures. **(B)** Related to Figure 8A. A representative snapshot of DEtail-seq signals showing ptDNA break patterns in Col-0 and *atrnh1c* under varying photoperiods.

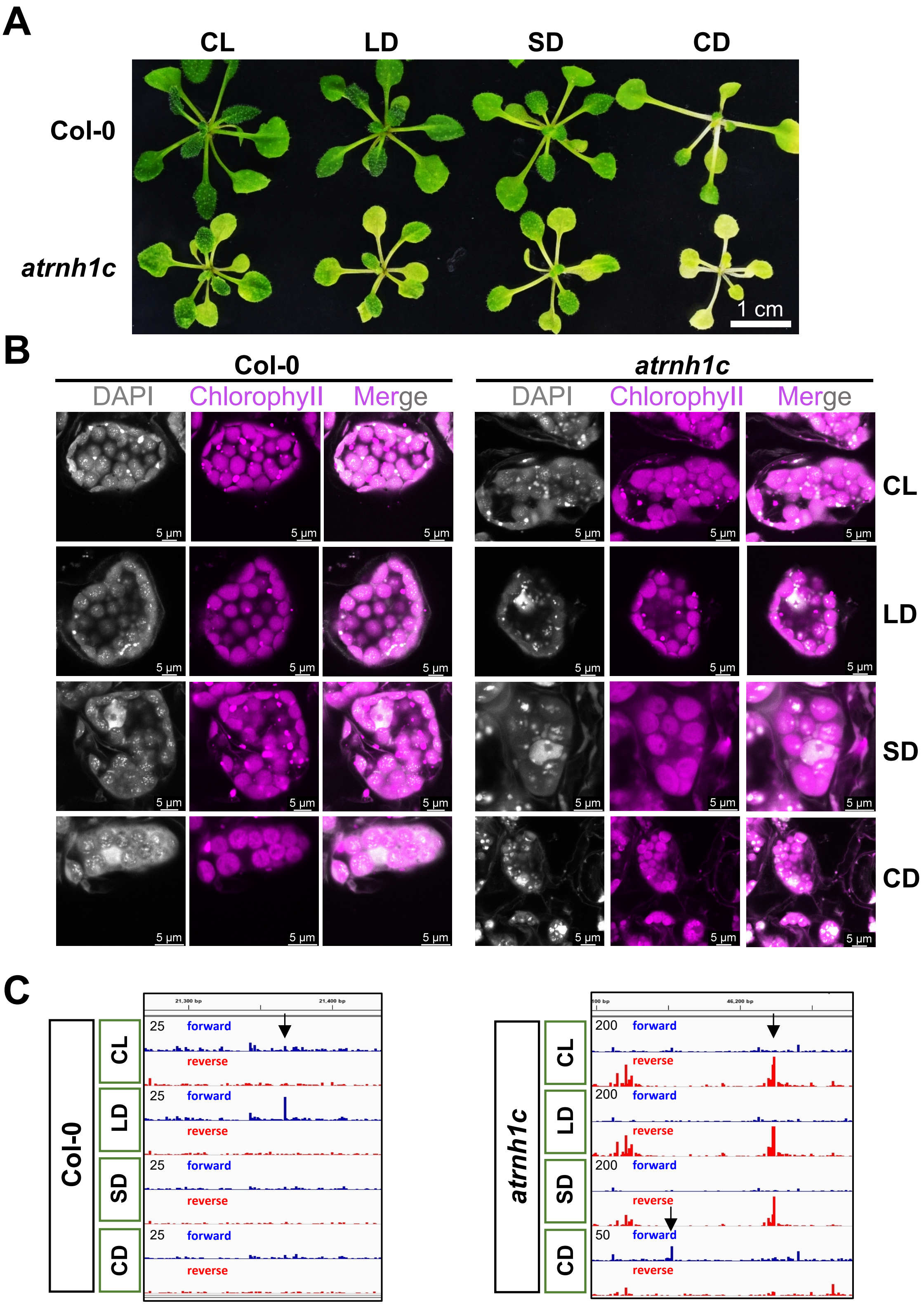

Figure S11. Morphology of chloroplast and nucleoid, and seedlings phenotype under different photoperiod treatments.

(A) Phenotype of seedlings grown under different photoperiods. Scale bars: 1 cm**. (B)** Morphology of chloroplasts and nucleoids in the seedlings described in **(A)**. **(C)** Representative images showing variation in break patterns in Col-0 and *atrnch1c* under different photoperiods. Arrowheads indicate sites of variation. CL (24 h Light), LD (16 h Light/8 h Dark), SD (8 h Light/16 h Dark), CD (24 h Dark).

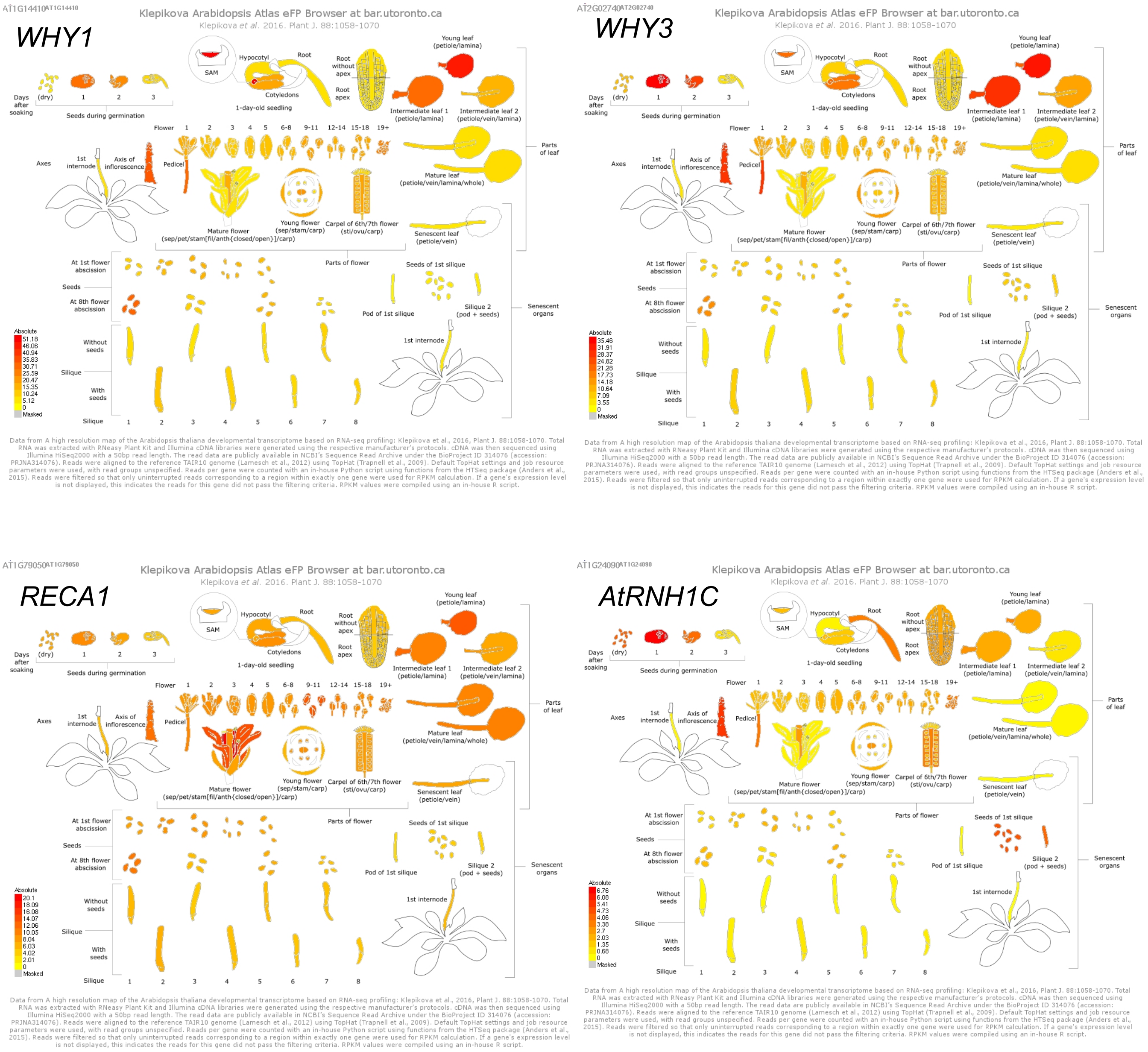

Figure S12. **The expression level of DNA repair genes**

Expression patterns of *WHY1*, *WHY3*, *RECA1*, and *AtRNH1C*. The data are from https://www.arabidopsis.org/.

Supplementary Table 1. Comparison of DNA break mapping methods.

Methods	DNA preparation	End process	Streptavidin-enrichment	PCR amplification	Quantification	DSB	SSB	Applications
BLESS	Fixed nuclei	Blunting	+	+	-	5’ end	-	Human, mouse
END-seq	Agarose-embedded	Blunting	+	+	-	5’ end	--	Human
DSB-capture	Fixed nuclei	Blunting	+	+	-	5’ end	-	Human
BLISS	Slide/ plate	Blunting	-	+	UMI	5’ end	-	Human, mouse
i-BLESS	Agarose-embedded	Blunting	+	+		5’ end	--	Yeast
qDSB-seq	Agarose-embedded	Blunting	+	+	Spike-in	5’ end	--	Human, yeast
TrAEL-seq	Agarose-embedded	-	+	+	-	3’ end	+	Human, yeast
INDUCE-seq	Fixed cells	Blunting	-	-	Cell number	5’ end	--	Human
GLEO-seq	*in vitro*	Denaturation	+	+	-	3’ end	++	Human, yeast
SSiNGLe	Fixed nuclei	MNase & denaturation	-	+/-	-	3’ end	++	Human, mouse
DEtail-seq	Agarose-embedded	-	-	+	Spike-in	3’ end	+++	Human, mouse, yeast, plant

Supplementary Table 2. Sequence of qPCR primers for detecting *I-CeuI* digestion efficiency.

Primer	Sequence
F1	GACAGTGCCCAGATCGTTACGCC
R1	CCATGTATGGGGGCTGACGCC
F2	GGTCACCAACTTCCTTAACCTTCCGGC
R2	CGGCAAAATAGCCCCGTAACTTCGG
F3	GGCAATGCAAGGGAAGCCATCGA
R3	TTCTCATGGATTTATTGGCGCTGCACT
